# On convergence for hybrid models of gene regulatory networks under polytopic uncertainties: a Lyapunov approach

**DOI:** 10.1007/s00285-021-01690-3

**Published:** 2021-11-18

**Authors:** Mirko Pasquini, David Angeli

**Affiliations:** 1 Department of Electrical and Electronic Engineering, Imperial College of London, London, UK; 2 Present Address: Division of Decision and Control, KTH Royal Institute of Technology, Stockholm, Sweden; 3 Competence Centre for Advanced Bioproduction by Continuous Processing, AdBIOPRO, Stockholm, Sweden; 4 Department of Information Engineering, University of Florence, Florence, Italy

**Keywords:** Gene regulatory networks, Lyapunov methods, Linear matrix inequalities, Convergence analysis, Systems biology, Polytopic uncertainties, 92C42, 93D30

## Abstract

Hybrid models of genetic regulatory networks allow for a simpler analysis with respect to fully detailed quantitative models, still maintaining the main dynamical features of interest. In this paper we consider a piecewise affine model of a genetic regulatory network, in which the parameters describing the production function are affected by polytopic uncertainties. In the first part of the paper, after recalling how the problem of finding a Lyapunov function is solved in the nominal case, we present the considered polytopic uncertain system and then, after describing how to deal with sliding mode solutions, we prove a result of existence of a parameter dependent Lyapunov function subject to the solution of a feasibility linear matrix inequalities problem. In the second part of the paper, based on the previously described Lyapunov function, we are able to determine a set of domains where the system is guaranteed to converge, with the exception of a zero measure set of times, independently from the uncertainty realization. Finally a three nodes network example shows the validity of the results.

## Introduction

In the last few years control theory tools have been extensively used in biology, to both understand natural biological systems or design new ones to perform specific tasks (Blanchini et al. 2018; Qian et al. 2018). Within a cell, in fact, multiple regulatory mechanisms coexist (Alon 2007; Freeman 2014) and among these, transcriptional regulation involving genes and transcription factors plays a crucial role. Transcription factors are proteins which can either activate or inhibit the transcription of different genes, which in turn can produce other transcription factors, determining a set of relations described by a gene regulatory network (GRN) (Alon 2007). Different modelling approaches are used to mathematically describe a GRN, depending on whether the analysis to perform is either quantitative or qualitative (Karlebach and Shamir 2008). Continuous models, derived from the study of chemical reaction networks and quasi-steady state approximation of faster dynamics, usually involve Hill functions to describe regulatory interactions and are particularly well suited for a quantitative analysis (Alon 2007; Le Novère and Nicolas 2015; Murray and Del Vecchio 2014). However, a quantitative approach may not always be possible as biological systems are inherently uncertain and the measurement of key quantities may be affected by noise or impossible to take; for these reasons, qualitative approaches have been developed in literature. Asynchronous boolean networks have been studied to analyse complex biological systems (see for example Tournier and Chaves 2009 and references therein), as many interactions and system quantities can be described by logic variables (e.g. HIGH or LOW concentration, protein production is ACTIVE or INACTIVE, etc.). Despite being a valid and simple method, completely discrete analysis can lose track of some dynamical behavior (Saadatpour and Albert 2016) and it may be necessary to consider hybrid models. The hybrid modelling approach was introduced in Glass and Kauffman (1973) and has been studied and adapted by many authors (see for example Casey et al. 2006; De Jong et al. 2003, 2004; Ropers et al. 2006; Cummins et al. 2016; Gedeon 2020). Based on the step approximation of steep Hill functions (Alon 2007), this approach gives rise to a piecewise affine (PWA) model. In Casey et al. (2006) the authors studied this model and gave stability conditions of equilibria based on a State Transition Graph (STG), a graph that qualitatively describes the system trajectories. The STG is unchanged for a large range of parameters, making this kind of analysis attractive when the system’s knowledge is partial. Despite being a valid tool, there are a few limitations in the use of STGs. These graphs in fact, besides introducing spurious qualitative trajectories (i.e. paths in the graph that do not correspond to trajectories of the original system) (Casey et al. 2006), do not give clear answers when cycles are present (Grognard et al. 2007), making it difficult to distinguish between damped oscillations and limit cycles. This motivates the development of tools that can fill these gaps, by complementing these qualitative tools, exploiting additional quantitative information from the system. One of the most common approach in this sense is to consider Lyapunov functions, which can help to discriminate between different kind of dynamical behaviors, otherwise indistinguishable from the graph, and the use of which is even suggested in the Conclusion of Casey et al. (2006), and which are the object of this work. The use of Lyapunov functions in the study of biochemical networks is well known in literature. In Ali Al-Radhawi and Angeli (2016) the authors studied chemical reaction networks stability and control, using piecewise linear in rates Lyapunov functions, in Blanchini and Giordano (2014) the authors employed piecewise linear Lyapunov functions to assess structural properties of biological networks, while in Chesi and Hung (2008) the authors, based on a linear matrix inequalities (LMIs) framework, used polynomial Lyapunov functions to study the stability of genetic regulatory networks with SUM regulatory functions. In our previous work (Pasquini and Angel 2019) we considered the aforementioned PWA model of a GRN and, using the model structure and the information from the STG, we developed an LMI framework to find a Lyapunov function for the system, in order to assess its convergence properties, even in the presence of cycles in the STG. In that context we assumed complete knowledge of the system’s parameters, an assumption that in general does not hold. However, Lyapunov methods are used also in the uncertain setting and a particular case is that of polytopic uncertainties for linear systems, in which the system’s matrix is the convex combination of a set of known matrices, with the weights of this combination being unknown. Two approaches are generally employed in literature. The first one consists in searching for a Lyapunov function which is common between all the systems obtained by considering the realizations associated with the vertices of the uncertainty polytope (Liberzon 2003; Lin and Antsaklis 2005). This approach, although allowing to conclude certain stability and stabilizability properties of the system, is challenging and can lead to a conservative solution, as it does not consider how the extremal behaviors are combined in any given uncertainty realization. The other approach, which we will consider in this work to deal with polytopic uncertainties, is to search for a parameter dependent Lyapunov function (PD-LF). In Gahinet et al. (1996) the authors first showed how to convexify the problem of finding an affinely parameter dependent Lyapunov function, through the solution of a set of LMIs. Following this many authors proposed different LMIs framework to deal with linear parameter varying systems stability (Chesi et al. 2004; Neto 1999; Oliveira and Peres 2006) and stabilizability (Lin and Antsaklis 2007; Zhai et al. 2003) through the use of PD-LFs, which are in general polynomially dependent on the uncertain parameters. Although the literature on parameter dependent lyapunov functions for polytopic uncertain systems is vast and still developing, many aspects are not usually considered. In most of the cases where both polytopic uncertainties and switching systems are considered, the problem of stabilizability is addressed (i.e. the choice of a switching signals to stabilise the system). However, in a PWA model of a GRN, the switching is state dependent and cannot be chosen. Moreover multistability, which is a common property of biological systems, is usually not considered; instead global asymptotic stability of a single equilibrium is a common theme in many of these works. For example in Arcak and Sontag (2008) the authors propose techniques based on dissipativity of individual gene subsystems in order to build separable Lyapunov functions that can be used to assess global asymptotic stability of the network. LMI conditions are used to infer the parameter ranges where such conditions can be fulfilled. In contrast, our paper does not focus on global asymptotic stability properties and exploits the structure of PWA models in order to build robust Lyapunov function for global convergence analysis.

In the present paper we consider polytopic uncertainties in piecewise affine models of GRNs, to address the issue that the system’s parameters may not be perfectly known (as in qualitative methods), but bounded within preassigned ranges. We provide an LMI framework whose solution describes a parameter dependent piecewise quadratic Lyapunov function (PD-PWQ-LF) for the system, i.e. a Lyapunov function that depends explicitly on the unknown parameters describing the particular uncertainty realization, which will allow us to describe a convergence set for the system, robust with respect to the uncertainty. Our analysis can deal with multistable systems, as information on equilibria location is not explicitly taken into consideration when defining the LMI framework. Moreover it is remarked that even if in literature there are results on parameter dependent Lyapunov functions with more complex and general structures (see for example Chesi et al. 2005), in this work we consider parameter dependent Lyapunov functions which are only affinely dependent on the system’s uncertain parameters, as these allow to deal in a straightforward manner with sliding mode monotonicity and continuity on the boundary of regulatory domains, as it will be clear in the following. The paper is structured as follows: in Sect. 2 some mathematical notation and preliminaries are defined, while in Sect. 3 the piecewise affine model and its differential inclusion extension are described, together with a brief recap of our work (Pasquini and Angel 2019) on piecewise quadratic Lyapunov functions for the nominal PWA model. In Sect. 4 the main contribution of the paper is presented: first we describe the considered polytopic uncertain model and the desired form of the Lyapunov function, then, after a discussion on the constraints that need to be enforced by this function, we give the conditions for its existence in the form of an LMI framework (Theorem 1). Finally we prove that, for any realization of the system uncertainty, the set where the system converges is contained in a computable set depending only on the vertices value of the uncertainty polytope. In Sect. 5 we give a numerical example showing the applicability and the validity of the results for a three nodes GRN, while Sect. 6 concludes the paper and give some possible directions for future developments.

## Mathematical background

Let $$v = \begin{bmatrix} v_1 \ldots v_n \end{bmatrix}^T$$ be a vector in ${\mathbb {R}}^n$. With $v \ge 0$ we mean that all the components of *v* are non-negative. The set of all $v \in {\mathbb {R}}^n$ such that $v \ge 0$ is denoted with ${\mathbb {R}}^n_+$. Let $W = \{w_1, \ldots , w_m\}$ be a set of vectors. We define the conic hull of *W* as ${\text {cone}(W) := \{v \in {\mathbb {R}}^n \ | \ \exists \alpha \in {\mathbb {R}}^m_+ \ s.t. \ v = \sum \nolimits _{i = 1}^m \alpha _i w_i \}}$. Let $S_m$ denote the standard simplex of dimension *m*, i.e. the set $S_m:=\{\alpha \in {\mathbb {R}}^n_+ | \sum \nolimits _{i=1}^m \alpha _i = 1\}$ .

We define the convex hull of *W* as $\text {conv}(W) := \{v \in {\mathbb {R}}^n \ | \ \exists \alpha \in S_m \ s.t. \ v = \sum \nolimits _{i = 1}^m \alpha _i w_i \}$.

A set-valued map $H : X \rightarrow 2^Y$ is a map that associates a point in *X* to a subset of *Y*.

With $\sum \nolimits _{v \in W}$ we denote the sum over the elements of the countable set *W*, while with $\bigcup \nolimits _{v' \in W'}$ we denote the union over the elements of the set $W'$.

Given a matrix $M \in {\mathbb {R}}^{n \times n}$, $M_{ij}$ denotes the element in its *i*-th row and *j*-th column. Let $M \in {\mathbb {R}}^{n \times n}$ be a symmetric matrix. We denote with $M \succ 0 (M \succeq 0)$ the fact that *M* is positive definite (semidefinite), and with $M \prec 0 (M \preceq 0)$ the fact that *M* is negative definite (semidefinite).

A polyhedron *P* in ${\mathbb {R}}^n$, is the set described by:

1 $$\begin{aligned} P = \{x \in {\mathbb {R}}^n \ s.t. \ A x \le b, A \in {\mathbb {R}}^{m \times n}, b \in {\mathbb {R}}^m\} \end{aligned}$$

Equation (1) is called the *H*-representation of *P*. Every polyhedron’s *H*-representation can be converted to a *V*-representation (Avis et al. 2002; Herceg et al. 2013; Iervolino et al. 2017b), namely:

2 $$\begin{aligned} P = \text {conv}\{V\} + \text {cone}\{R\} \end{aligned}$$

where $$V = \begin{bmatrix}v_1 \ldots v_\nu \end{bmatrix}$$ denotes the set of vertices of *P*, and $$R = \begin{bmatrix} r_1 \ldots r_\rho \end{bmatrix}$$ the set of its rays, with the notation in (2) meaning that any element in *P* can be expressed as the sum of an element belonging to $\text {conv}\{V\}$ and an element belonging to $\text {cone}\{R\}$. Any polyhedron *P* in ${\mathbb {R}}^n$ can be embedded in an higher dimensional cone, called the homogenization cone, i.e. the cone:

$$\begin{aligned} {\hat{C}}_P := \text {cone}\{{\bar{v}}_1,\ldots ,{\bar{v}}_\nu ,{\bar{r}}_1, \ldots {\bar{r}}_\rho \} \end{aligned}$$

where:

$$\begin{aligned} {\bar{v}}_i = \begin{bmatrix} v_i \\ 1 \end{bmatrix} \qquad {\bar{r}}_i = \begin{bmatrix} r_i \\ 0 \end{bmatrix} \end{aligned}$$

The homogenization cone of *P* has the property that *P* can be obtained as the intersection of the cone with an hyperplane ${\mathscr {H}}$ in ${\mathbb {R}}^{n+1}$, thus allowing to give sufficient conditions which guarantee a chosen quadratic function to be either positive or negative definite (semidefinite), inside the original polyhedron *P*.

In particular we recall the following property from Iervolino et al. (2017a), which we also used in Pasquini and Angel (2019), to express sign definiteness conditions of quadratic functions inside a polyhedron *P*.

Consider the quadratic function:

3 $$\begin{aligned} x^T M x + 2 x^T v + \omega = {\bar{x}}^T {\overline{M}} {\bar{x}} \end{aligned}$$

where:

$$\begin{aligned} {\bar{x}} = \begin{bmatrix} x \\ 1 \end{bmatrix}, \quad {\overline{M}} = \begin{bmatrix} M &{}\quad v \\ v^T &{}\quad \omega \end{bmatrix} \end{aligned}$$

Let *P* be a polyhedron and ${\hat{C}}_P$ its homogenization cone, defined above. Then it holds:

4 $$\begin{aligned} \varGamma ^T {\overline{M}} \varGamma + N \preceq 0 \Rightarrow {\overline{M}} \preceq _{{\hat{C}}_P} 0 \end{aligned}$$

where $\varGamma$ is the matrix whose columns are the rays of the cone ${\hat{C}}_P$, *N* is any entrywise non-negative matrix and ${\overline{M}} \preceq _{{\hat{C}}_P} 0$ means that the quadratic function (3) is non-positive for any $x \in {\hat{C}}_{P}$, and consequently in the polyhedron *P*. For further details the interested reader is referred to Iervolino et al. (2017a).

## Hybrid models of GRNs and Lyapunov functions

### Hybrid model

*In this section the hybrid model considered to describe the GRN dynamics is presented. This section is mainly recalled from* (Casey et al. 2006; De Jong et al. 2004; Pasquini and Angeli 2018), *to which the reader is referred for a more comprehensive discussion*

Let $C \in {\mathbb {R}}^{n \times n}$ be a diagonal matrix with positive entries and let $f : {\mathbb {R}}^n_+ \rightarrow {\mathbb {R}}^n_+$ be a piecewise constant function, defined on a box partition of the positive orthant (partition that will be characterised below). We consider the following hybrid model:

5 $$\begin{aligned} {\dot{x}} = f(x) - C x, \qquad x \in {\mathbb {R}}^n_+ \end{aligned}$$

where $$x = \begin{bmatrix} x_1&\ldots&x_n \end{bmatrix}^T\in {\mathbb {R}}^n_+$$ represents the protein concentration vector (with $x_i$ being the concentration of the protein $P_i$), $C = \text {diag}(c_1, \ldots , c_n)$, with $c_i > 0$, represents the degradation rates matrix and $$f(x) = \begin{bmatrix} f_1(x)&\ldots&f_n(x) \end{bmatrix}^T$$ represents the production rate function, sometimes referred to as the regulation function.

Let any axis ${\mathscr {X}}_i$ of the positive orthant, be partitioned as:

6 $$\begin{aligned} {\mathscr {X}}_i = \{[0,\theta _{i,1}), \{\theta _{i,1}\}, (\theta _{i,1},\theta _{i,2}), \ldots (\theta _{i,m_i},+\infty )\} \end{aligned}$$

The $\theta$s in (6) are called thresholds. Such partition divides the state space in open boxes, inside which we consider the regulation function *f*(*x*) to be constant. These boxes are called regulatory domains, as opposed to the ones called switching domains, in which at least one of the state variables takes on a threshold value. We define the order of a switching domain $D_s$ as the number of switching variables in $D_s$ i.e. state variables that are on one of their threshold values.

The set of regulatory domains is denoted by ${\mathscr {D}}_R$, while the set of switching domains is denoted by ${\mathscr {D}}_S$. Inside each regulatory domain *D* the production function is considered constant and indicated as $f_D$, hence the dynamics is affine, with the property that any trajectory monotonically converges towards the focal point $\phi (D) = C^{-1} f_D$ and, if this is not in $\text {cl}(D)$, then the trajectory will eventually leave the domain (Casey et al. 2006).

#### Remark 1

The above definition of the production function for system (5), is a consequence of the ON–OFF approximation of the gene input functions (Alon 2007). In fact in works like Casey et al. (2006) and De Jong et al. (2004), and our previous work Pasquini and Angeli (2018), the production function is considered as a combination of step functions, the thresholds of which give rise to the same box partition described above defining, de facto, a piecewise constant function. In Plahte et al. (1998) the authors gave a general framework which allows to describe any logical function with the use of steps or sigmoids functions. $\blacktriangleleft$

The switching domains are the zero measure sets where the production rate function is not uniquely defined and so a further construction due to Filippov (1988) is used. In particular the system is extended to a differential inclusion:

7 $$\begin{aligned} {\dot{x}} \in F(x) \end{aligned}$$

for which:

8 $$\begin{aligned} F(x) = {\left\{ \begin{array}{ll} \begin{array}{ll} \{ f_D - C x \} &{}\quad \text {if} \;x \in D \in {\mathscr {D}}_R\\ \text {conv} \{f_{D'} - C x,\ D' \in R(D)\} &{}\quad \text {if} \;x \in D \in {\mathscr {D}}_S \end{array} \end{array}\right. } \end{aligned}$$

in which *R*(*D*) denotes the set of regulatory domains adjacent to the switching domain *D*. A solution (in the sense of Filippov) of system (5) is an absolutely continuous function $x(\cdot )$, satisfying the differential inclusion (7), for almost every *t*, with the set-valued map *F* defined as in (8). It is possible that a solution $x(\cdot )$ of the differential inclusion (7), lays for a certain amount of time on a switching domain (i.e. on the surface of discontinuity of *f*(*x*)), leading to a so called sliding mode solution.

As explained in Casey et al. (2006) and De Jong et al. (2003, 2004), it is possible to define a state transition graph of the PWA system, a graph that qualitatively characterise the system trajectories in relation to the domains (both regulatory and switching). This construct will appear in a later assumption (Assumption 1), but for more details on how the STG can be constructed and used, the interested reader is referred to the aforementioned literature.

We now introduce a simple example that will help clarify the above notation.

### Example: toggle switch

**Fig. 1:**
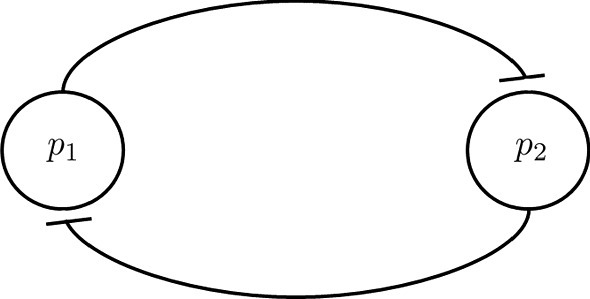
Gene regulatory network of a toggle switch system

Consider the following piecewise affine model of a toggle switch (the GRN of which is depicted in Fig. 1):

9 $$\begin{aligned} {\left\{ \begin{array}{ll} {\dot{x}}_1 = b_{10} + b_{11} s^-(x_2, \theta _2) - c_1 x_1\\ {\dot{x}}_2 = b_{20} + b_{21} s^-(x_1, \theta _1) - c_2 x_2\\ \end{array}\right. } \end{aligned}$$

where $s^-(x_i,\theta _j)$ is the step function:

10 $$\begin{aligned} s^-(x_i, \theta _j) = {\left\{ \begin{array}{ll} \begin{array}{rl} 1 &{}\quad \text {if}\; x_i < \theta _j \\ 0 &{}\quad \text {if}\; x_i > \theta _j \end{array} \end{array}\right. } \end{aligned}$$

and $$b_{10} = 0.02$$, $$b_{20} = 0.08$$, $$b_{11} = 3$$, $$b_{21} = 3.5$$, $$c_1 = 0.7$$, $$c_2 = 1.1$$, $$\theta _1 = \theta _2 = 2$$.

The toggle switch is one of the simplest gene regulatory networks to consider [and one of the first networks that has been built synthetically (Collins et al. 2000)], in which two proteins $P_1$ and $P_2$ act as each other inhibition transcription factor. For certain sets of parameters, the system is known to show bistability, as the two stable configurations are when one of the two proteins concentration is high, while the other one is low.

**Fig. 2:**
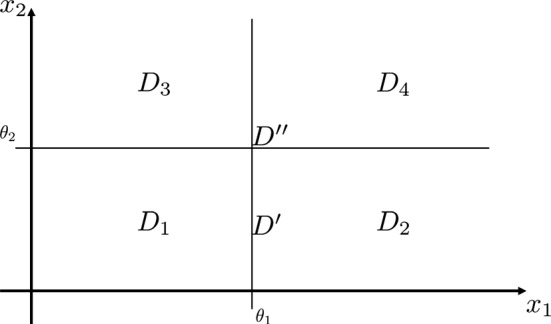
Positive orthant partition for systems (9)

**Fig. 3:**
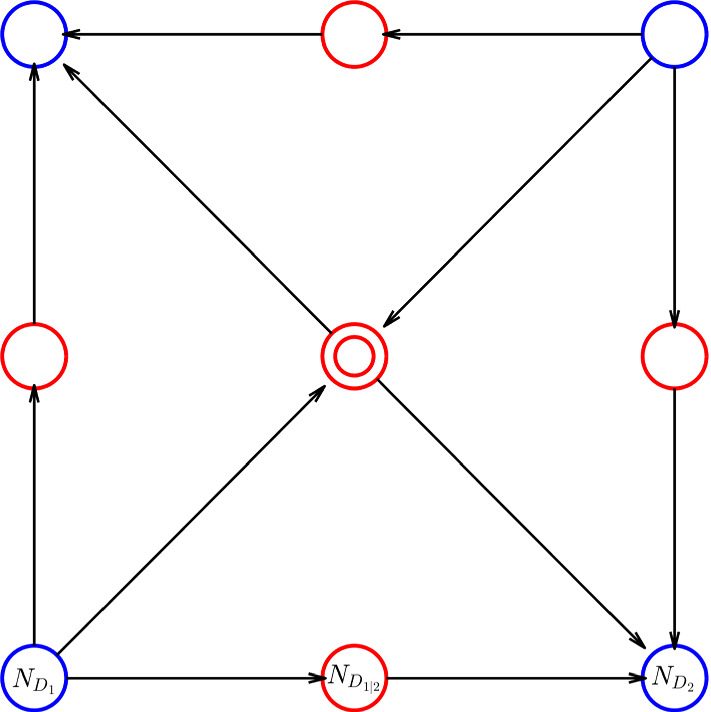
State transition graph of system (9) (color figure online)

**Fig. 4:**
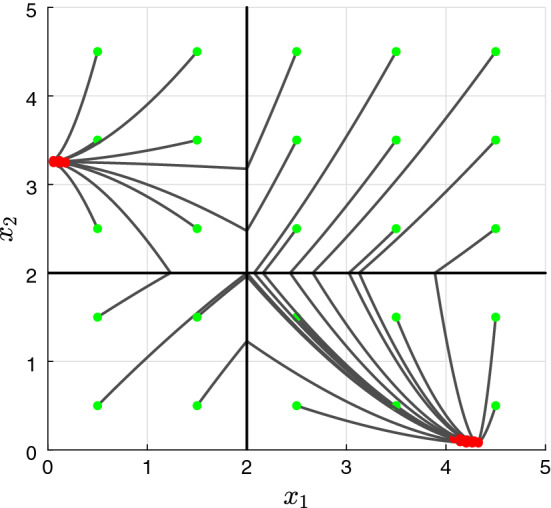
Trajectories of system (9). Red dots represent end points of the trajectories, while the green ones represent their initial conditions (color figure online)

As can be seen in Fig. 2, the thresholds $\theta _1$ and $\theta _2$ give rise to four regulatory domains (i.e. $D_1$, $D_2$, $D_3$, $D_4$), and five switching domains: four of which of order 1 (e.g. the domain $D' = \partial D_1 \cap \partial D_2$) and one of order 2 (i.e. $D'' = \partial D_1 \cap \partial D_2 \cap \partial D_3 \cap \partial D_4$). The procedure described in De Jong et al. (2004) generates the state transition graph in Fig. 3. In this graph: the blue nodes are associated to regulatory domains, while the red ones are associated to switching domains (one circle: domains of order 1, two circles: domains of order 2). It is clear from it that the system exhibits bistability, property that is also confirmed by the trajectories in Fig. 4.

The intersection between the closure of the four regulatory domains (i.e. the point $(\theta _1,\theta _2)$) is a singular equilibrium—in the sense that $0 \in F(\theta _1,\theta _2)$, with *F*(*x*) being the set-valued map defined in (8)

### Piecewise quadratic Lyapunov function

Lyapunov functions are a tool that is extensively used in control theory and the study of dynamical systems, allowing the study of stability and convergence properties (Khalil 2002). In Pasquini and Angeli (2018) we presented an LMI framework to find, if one exists, a Piecewise Quadratic Lyapunov function (PWQ-LF) for system (5), which can be formally proved to be eventually non-increasing along any system trajectory (Pasquini and Angel 2019). In particular the Lyapunov function *V* is defined as:

11 $$\begin{aligned} \begin{aligned} V(x)&= V_D(x) \qquad \text {if }\ x \in D, \ D \in {\mathscr {D}}_R \\ V_D(x)&= x^T P_D x + 2 d_D^T x + \omega _D \\&= \begin{bmatrix} x^T&1 \end{bmatrix} \begin{bmatrix} P_D &{}\quad d_D\\ d_D^T &{}\quad \omega _D \end{bmatrix} \begin{bmatrix} x \\ 1 \end{bmatrix} = {\bar{x}}^T {\overline{P}}_D {\bar{x}} \end{aligned} \end{aligned}$$

and it is asked to satisfy the following constraints:

12 $$\begin{aligned}&\nabla V_D(x) \cdot (f_D - C x) \le 0, \;\; \forall x \in D,\ \ \forall D \in {\mathscr {D}}_R \end{aligned}$$

13 $$\begin{aligned}&\overline{\nabla V}_D(x) \cdot f \le 0,\;\; \forall x \in D,\;\; \forall D \in {\mathscr {D}}_S, \\&\quad \forall f \in \text {conv} \{f_{D'} - C x, D' \in R(D)\} \bigcap T_x D\nonumber \end{aligned}$$

14 $$\begin{aligned}&\lim _{\begin{array}{c} y \rightarrow x, \\ y \in D \end{array}} V_D(y) = \lim _{\begin{array}{c} y' \rightarrow x, \\ y' \in D' \end{array}} V_{D'}(y') , \;\; \forall x \in D_s, \\&\quad \forall D, D' \in R(D_s) , \nonumber \\&\quad \forall D_s \in \mathscr {CD}\nonumber \end{aligned}$$

where $T_x D$ is the tangent cone to *D* in *x*, $\overline{\nabla {V}}_D$ represents the gradient of the function $V_{{\hat{D}}}$, with ${\hat{D}}$ being any regulatory domain adjacent to *D*, and $\mathscr {CD}$ denotes the set of switching domains on which we asked the function *V* to be continuous, namely the ones for which the set of sliding mode directions is non-empty and the ones associated to cycles in the State Transition Graph.

To summarise: constraints (12) and (13) are relative to the monotonicity of the Lyapunov function in regulatory domains and switching domains with sliding modes respectively, while (14) refers to the continuity of the Lyapunov function in particular switching domains—the set of which is referred as $\mathscr {CD}$.

These constraints are enforced through a set of LMIs, which is omitted here for the sake of space and ease of explanation, however, for further details on the description and derivation of this set, the interested reader is referred to our previous works (Pasquini and Angel 2019; Pasquini and Angeli 2018). In particular it can be proven that, for a function *V* satisfying (12)–(14), the following property holds:

#### Proposition 1

Consider the system (5) and let $V : {\mathbb {R}}^n_+ \rightarrow {\mathbb {R}}$ be a Lyapunov function satisfying (12)–(14). Then:

15 $$\begin{aligned} \lim \limits _{\tau \rightarrow \infty } \mu \left( \left\{ t \ge \tau : {d V(x(t)) \over d t} < -\varepsilon \right\} \right) = 0 , \qquad \forall \varepsilon > 0 \end{aligned}$$

where $\mu (S)$ denotes the Lebesgue measure of the set *S*.

In the following we refer to property (15) as a result of convergence to zero of ${dV \over dt}(x(t))$ in the sense of measure.

In words, condition (15) states that, given an arbitrarily small $\varepsilon > 0$ and a set $\varOmega$ where the solution $x(\cdot )$ is contained [e.g. $\varOmega = {\mathbb {R}}^n_+$ or $\varOmega = {\mathscr {B}}$, with ${\mathscr {B}}$ being a positively invariant set with respect to (5)], then as $t \rightarrow \infty$ the solution will be almost surely (in the sense of Lebesgue measure) in the set $\varOmega {\setminus } \{x \in \varOmega \ |\ {\dot{V}}(x) < -\varepsilon \}$. This means that, by taking values of $\varepsilon$ converging to 0, we can infer information on the convergence set for the system.

#### Remark 2

In Pasquini and Angel (2019) a few more technicalities are introduced to avoid the convergence to a set where the Lyapunov function is not defined. This has been done exploiting a construction—called natural extension of the Lyapunov function—which is equal to the original function *V* almost everywhere. More details can be found in the aforementioned paper. $\blacktriangleleft$

The existence of a feasible solution to this set of LMIs, and consequently of a Lyapunov function for the system, is strictly dependent on the system parameters, which are often highly uncertain due to the nature of biological systems. In the next Section the case of uncertainties on the production rate function is considered.

## Main contribution

We now introduce polytopic uncertainties on the production rate function, to model the fact that its exact value is unknown and possibly subject to variability.

Let $C \in {\mathbb {R}}^{n \times n}$ be a diagonal matrix with positive diagonal entries and let $f^1(x), \ldots , f^L(x)$ be *L* piecewise constant production functions, as defined in Sect. 3. Consider the system $\varSigma _k$, defined as:

16 $$\begin{aligned} \varSigma _k : {\dot{x}} = f^k(x) - C x, \quad k \in \{1,\ldots ,L\}, \quad x \in {\mathbb {R}}^n_+ \end{aligned}$$

$\varSigma _k$ is called an extremal system. Let ${\mathscr {U}}(f^1,\ldots ,f^L)$ be the set of systems:

17 $$\begin{aligned} {\mathscr {U}}(f^1,\ldots ,f^L) := \{\sigma ^\lambda : {\dot{x}} = f^\lambda (x) - C x, \ x \in {\mathbb {R}}^n_+\} \end{aligned}$$

in which:

18 $$\begin{aligned} f^\lambda (x) := \sum \limits _{k = 1}^L \lambda _k f^k(x), \qquad \lambda \in S_L \end{aligned}$$

and $S_L$ is the standard simplex of dimension *L*.

In the following we will use the shortened notation:

$$\begin{aligned} {\mathscr {U}}_1^L \equiv {\mathscr {U}}(f^1,\ldots ,f^L) \end{aligned}$$

as the dependence of the set from the considered functions $f^1, \ldots , f^L$ is implicit.

From now on the following Assumption is considered to be satisfied:

### Assumption 1

All extremal systems $\varSigma _1, \ldots , \varSigma _L$ have the same State Transition Graph (STG) and the same thresholds. $\blacktriangleleft$

### Remark 3

The first part of Assumption 1 is not too restrictive, in the sense that the STG is generally unchanged under a large range of parameters (see Casey et al. 2006; De Jong et al. 2004), and assuming that the STG is the same for all extremal systems means that these are “qualitatively” similar, which is the starting point of this analysis. If multiple graphs arise one could, in principle, attempt a partition of the uncertainty set, so as to recover Assumption 1 on each of its element.

On the other hand to consider that thresholds are unchanged among all extremal systems can be artificial. However, considering uncertain thresholds, entails a structural change in the positive orthant partition, with remarkable difficulties in imposing continuity constraints—which will potentially convert in non-linear constraints, given how condition (14) is ultimately enforced (see (Pasquini and Angel 2019, Eq. (43)) or Eq. (42)–(44) of this work). We recognize the need to address this matter in potential future research. $\blacktriangleleft$

### Example: toggle switch with polytopic uncertainties

To better clarify the new notation introduced above, consider again the toggle switch example of Sect. 3.2, but this time allow $b_{11} \in [b_{11}^-,\ b_{11}^+]$ and $b_{21} \in [b_{21}^-,\ b_{21}^+]$, with $b_{11}^- = b_{21}^- = 2.5$ and $b_{11}^+ = b_{21}^+ = 5$.

We can define the four extremal systems:

$$\begin{aligned} \varSigma _1&: {\left\{ \begin{array}{ll} {\dot{x}}_1 = b_{10} + b_{11}^- s^-(x_2, \theta _2) - c_1 x_1\\ {\dot{x}}_2 = b_{20} + b_{21}^- s^-(x_1, \theta _1) - c_2 x_2\\ \end{array}\right. } \varSigma _2&: {\left\{ \begin{array}{ll} {\dot{x}}_1 = b_{10} + b_{11}^+ s^-(x_2, \theta _2) - c_1 x_1\\ {\dot{x}}_2 = b_{20} + b_{21}^- s^-(x_1, \theta _1) - c_2 x_2\\ \end{array}\right. } \\ \varSigma _3&: {\left\{ \begin{array}{ll} {\dot{x}}_1 = b_{10} + b_{11}^- s^-(x_2, \theta _2) - c_1 x_1\\ {\dot{x}}_2 = b_{20} + b_{21}^+ s^-(x_1, \theta _1) - c_2 x_2\\ \end{array}\right. } \varSigma _4&: {\left\{ \begin{array}{ll} {\dot{x}}_1 = b_{10} + b_{11}^+ s^-(x_2, \theta _2) - c_1 x_1\\ {\dot{x}}_2 = b_{20} + b_{21}^+ s^-(x_1, \theta _1) - c_2 x_2\\ \end{array}\right. } \end{aligned}$$

It is easy to verify (see Casey et al. 2006 for example) that all extremal systems share the same STG. The set ${\mathscr {U}}_1^4$, of possible system realizations given the uncertainty, can be rewritten in a parametrized form as:

$$\begin{aligned} \sigma ^\lambda : {\left\{ \begin{array}{ll} {\dot{x}}_1 = b_{10} + \big [(\lambda _1 + \lambda _3)b_{11}^- + (\lambda _2+\lambda _4) b_{11}^+\big ] s^-(x_2, \theta _2) - c_1 x_1\\ {\dot{x}}_2 = b_{20} + \big [(\lambda _1 + \lambda _2)b_{21}^- + (\lambda _3+\lambda _4) b_{21}^+\big ] s^-(x_1, \theta _1) - c_2 x_2\\ \end{array}\right. } \end{aligned}$$

with $\lambda \in S_4$, or equivalently:

$$\begin{aligned} {\left\{ \begin{array}{ll} {\dot{x}}_1 = b_{10} + \big [(1-\eta _1)b_{11}^- + \eta _1 b_{11}^+\big ] s^-(x_2, \theta _2) - c_1 x_1\\ {\dot{x}}_2 = b_{20} + \big [(1-\eta _2)b_{21}^- + \eta _2 b_{21}^+\big ] s^-(x_1, \theta _1) - c_2 x_2\\ \end{array}\right. } \end{aligned}$$

with $$\eta _i \in [0,1]$$, for $$i = \{1,2\}$$.

**Fig. 5:**
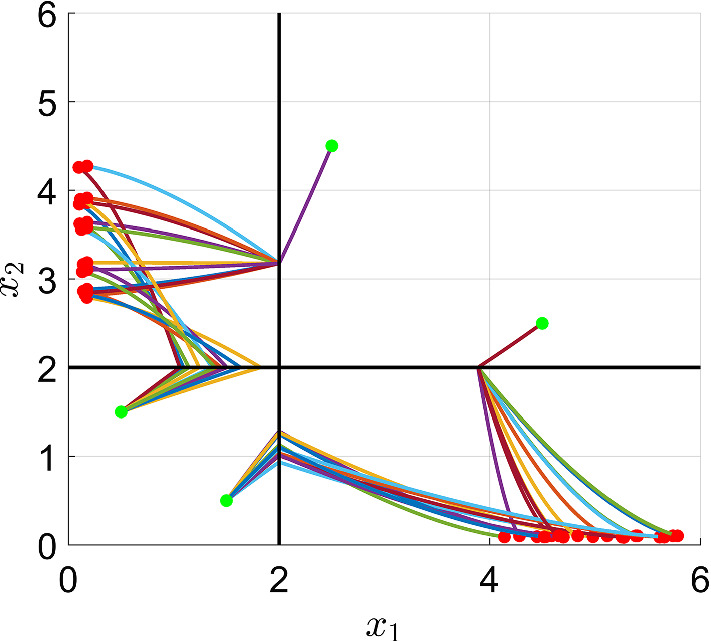
Trajectories of different toggle switch realizations for the example of Sect. 4.1, with polytopic uncertainties on the regulation functions, are considered

By varying $\lambda \in S_4$ (or $\eta \in S_1 \times S_1$) we obtain different realizations $\sigma ^\lambda \in {\mathscr {U}}_1^4$, with the two stable equilibria moving depending on such parameters, while saddle point $(\theta _1,\theta _2)$ remains unchanged (following the structure of the system and Assumption 1). In Fig. 5, trajectories for different realizations $\sigma ^\lambda \in {\mathscr {U}}_1^4$ are shown, starting from the same initial points.

$$\blacktriangleleft $$

In the following we will prove that we can define a set of linear matrix inequalities (LMIs) that, if satisfied, guarantees the existence of a Parameter Dependent Piecewise Quadratic Lyapunov Function (PD-PWQ-LF) of the form:

19 $$\begin{aligned} V^\lambda (x) = \sum \limits _{k = 1}^L \lambda _k V^k(x) \end{aligned}$$

where $V^k(x)$ is a particular PWQ-LF for the k-th extremal system $\varSigma ^k$, will be called an extremal Lyapunov function and will assume the form (11). Formally speaking, given a certain $\lambda \in S_L$, $V^\lambda (x)$ is a PWQ-LF for the system $\sigma ^\lambda \in {\mathscr {U}}^1_L$, and this will hold for all the $\lambda \in S_L$.

The set of LMIs to satisfy will take into account: the conditions to make $V^k(x)$ an extremal Lyapunov function for $\varSigma ^k$ and the conditions to guarantee that $V^\lambda (x)$ is a PWQ-LF for $\sigma ^\lambda$.

#### Remark 4

Asking for $V^k(x)$ to be an extremal Lyapunov function for the system $\varSigma ^k$, corresponds to asking (12)–(14) for $V^k$, with respect to $\varSigma ^k$ , with the only exception of the constraints on monotonicity along sliding modes (13), that should be substituted as explained in the next section. This is due to the fact that asking for $V^k$ to be non-increasing along any sliding mode solution of $\varSigma ^k$, is not enough to guarantee that $V^\lambda$ in (19) will be non-increasing along any sliding mode solutions of $\sigma ^\lambda$. $\blacktriangleleft$

### Sliding mode directions description

Consider a switching domain $D_s$. For any system in ${\mathscr {U}}_1^L$, any potential sliding mode direction *f* on $D_s$, can be expressed as:

20 $$\begin{aligned} f = \sum \limits _{D' \in R(D_s)}\sum \limits _{k=1}^L \gamma _{D'}^k f_{D'}^k - C x \end{aligned}$$

where $\gamma \in S_{L \cdot q}$ and $q := |R(D_s)|$. The $\gamma$s in (20) are the weights of a convex combination that takes into account all extremal systems, in order to generate candidate sliding mode directions at $x \in D_s$.

Let $I_{D_s}$ be the set of switching variables in $D_s$ (i.e. the variables that are on their thresholds in $D_s$).

The following polyhedron ${\mathscr {P}}_{D_s}$ can be defined:

21 $$\begin{aligned} {\mathscr {P}}_{D_s} := \left\{ \gamma \in S_{L \cdot q} : \begin{bmatrix}\overline{F^1}&\overline{F^2}&\ldots&\overline{F^L} \end{bmatrix} \gamma = \begin{bmatrix} {\bar{c}}\end{bmatrix} \right\} \end{aligned}$$

where ${\bar{c}}$ is a vector, the i-th component of which is:

22 $$\begin{aligned}{}[{\bar{c}}]_i = c_i \theta _{i,k}, \qquad i \in I_{D_s} \end{aligned}$$

where $c_i$ is the degradation rate of the protein $P_i$ and $\theta _{i,k}$ is the threshold value assumed by $x_i$ in the switching domain $D_s$ [see axis partition (6)], while $\overline{F^k}$ is the matrix:

23 $$\begin{aligned} \overline{F^k} = \begin{bmatrix} \big (f^k_{D_{i_1}}\big )^{I_{D_s}}&\ldots&\big (f^k_{D_{i_q}}\big )^{I_{D_s}} \end{bmatrix} \end{aligned}$$

in which $(f^k_D)^{I_{D_s}}$ is the vector obtained by selecting only the components from $(f^k_D)$, indexed by $I_{D_s}$, and $\{D_{i_1}, \ldots , D_{i_q}\}$ is the set of regulatory domains adjacent to $D_s$. ${\mathscr {P}}_{D_s}$ contains all the $\gamma$s that give rise to sliding mode directions and, by construction, it is the same among all the extremal systems and all the systems in ${\mathscr {U}}_1^L$. Being ${\mathscr {P}}_{D_s}$ a subset of the standard simplex $S_{L \cdot q}$, it is bounded and so can be written as:

24 $$\begin{aligned} {\mathscr {P}}_{D_s} := \text {conv}\{w_1, w_2, \ldots , w_{v_\gamma }\} \end{aligned}$$

Following an approach similar to the one we used in Pasquini and Angeli (2018), if every extremal Lyapunov function $V^k$ is monotone along the directions obtained by selecting the $\gamma$s in ${\mathscr {P}}_{D_s}$, then the function $V^\lambda$ will be monotone along any sliding modes solution.

The above property can be satisfied, by enforcing the following set of LMIs for any extremal LF $V^k$:

25 $$\begin{aligned} \varGamma _{D_s}^T L^k_{D_s,j} \varGamma _{D_s} + M^k_{D_s} \preceq 0, \qquad \forall j \in \{1, \ldots , \nu _\gamma \} \end{aligned}$$

where $\varGamma _{D_s}$ is the ray matrix of the homogenization cone ${\hat{C}}_{D_s}$ of $D_s$, $M^k_{D_s}$ is any entrywise non-negative and symmetric matrix and:

26 $$\begin{aligned} L^k_{D_s,j} = \begin{bmatrix} -2 P_D^k C &{}\quad P_D^k F w_j - C d^k_D \\ w_j^T {F}^T P^k_D - {d^k}_D^T C &{}\quad 2 {d^k}_D^T F w_j \end{bmatrix} \end{aligned}$$

where $P_D^k$ and $d_D^k$ are the matrices $P_D$ and $d_D$ in (11), for the extremal LF $V^k$, $w_j$ is the *j*-th vertex of ${\mathscr {P}}_{D_s}$ and *F* is the matrix defined as:

27 $$\begin{aligned} F = \begin{bmatrix} F^1&\ldots&F^L \end{bmatrix} \end{aligned}$$

with:

28 $$\begin{aligned} F^k = \begin{bmatrix} f^k_{D_{i_1}}&\ldots&f^k_{D_{i_q}} \end{bmatrix} \end{aligned}$$

where $\{D_{i_1}, \ldots , D_{i_q}\}$ is the set of regulatory domains adjacent to $D_s$.

#### Remark 5

The above approach is conservative, in the sense that not every $\gamma \in {\mathscr {P}}_{D_s}$ generate an actual sliding mode direction for the systems in ${\mathscr {U}}_1^L$, but ${\mathscr {P}}_{D_s}$ surely contains all of them. $\blacktriangleleft$

### Existence of a PD-PWQ-LF

The following Theorem gives the conditions to construct a PD-PWQ-LF of the form (19).

#### Theorem 1

Let $\varSigma _1, \ldots , \varSigma _L$ be *L* extremal systems as defined in (16). Let $V^1, \ldots , V^L$ be their extremal LFs, each one satisfying the LMIs guaranteeing (12) and (14) and the set of LMIs (25). Let, for any regulatory domain $D \in {\mathscr {D}}_R$:

29 $$\begin{aligned} \begin{aligned} \varGamma _D^T \delta {\tilde{P}}_D^{kj} \varGamma _D + M^{kj} \preceq 0, \qquad&k \in \{1,\ldots ,L-1\} \\&j \in \{i+1,\ldots ,L\} \end{aligned} \end{aligned}$$

in which $M^{kj}$ is any non-negative entrywise symmetric matrix, $\varGamma _D$ is the ray matrix of the homogenization cone ${\hat{C}}_D$ and:

30 $$\begin{aligned} \delta {\tilde{P}}_D^{kj} = \begin{bmatrix} 0 &{}\quad -\delta P_D^{kj} \delta f_D^{kj} \\ -(\delta f_D^{kj})^T \delta P_D^{kj} &{}\quad -2 (\delta d_D^{kj})^T \delta f_D^{kj} \end{bmatrix} \end{aligned}$$

where:

31a $$\begin{aligned}&\delta P_D^{kj} := P_D^j - P_D^k \end{aligned}$$

31b $$\begin{aligned}&\delta d_D^{kj} := d_D^j - d_D^k \end{aligned}$$

31c $$\begin{aligned}&\delta f_D^{kj} := f_D^j - f_D^k \end{aligned}$$

Let $$\sigma ^\lambda \in {\mathscr {U}}_1^L$$. Then:

32 $$\begin{aligned} V^\lambda (x) = \lambda _1 V^1(x) + \cdots + \lambda _L V^L(x) \end{aligned}$$

is a PWQ-LF for $\sigma ^\lambda$.

#### Proof

Fix a $\lambda \in S_L$. The system $\sigma ^\lambda$ is a PWA system and for $V^\lambda$ to be a PWQ-LF for $\sigma ^\lambda$, it needs to satisfy the constraints (12), (13) and (14).

$V^\lambda$ is a convex combination of the extremal Lyapunov functions and, given Assumption 1, (14) is naturally satisfied for $V^\lambda$.

Moreover, given (25), every extremal Lyapunov function is monotone along every sliding direction of $\sigma ^\lambda$, for any switching domain $D_s$, because of the construction of ${\mathscr {P}}_{D_s}$, and this guarantee that $V^\lambda$ satisfies (13) as well. We now only need to prove that (12) is satisfied for $V^\lambda$ with respect to $\sigma ^\lambda$. Let *D* be a regulatory domain of (5). Then:

33 $$\begin{aligned} \begin{aligned} {{\dot{V}}_D^\lambda }&= \nabla V_D^\lambda \big (f_D^\lambda - C x\big ) \\ {}&= \sum \limits _{k=1}^L \lambda _k \nabla V_D^k \left( \sum \limits _{j=1}^L \lambda _j f_D^j - C x\right) \\&= \sum \limits _{k=1}^L \lambda _k^2 {\dot{V}}_D^k + \sum \limits _{k=1}^L \sum \limits _{\begin{array}{c} j = 1 \\ j\ne k \end{array}}^L \lambda _k \lambda _j dV_D^{kj} \end{aligned} \end{aligned}$$

in which:

34 $$\begin{aligned} dV_D^{kj} := \nabla V_D^k \big (f_D^j - C x\big ) \end{aligned}$$

We can rewrite (33) as:

35 $$\begin{aligned} {{\dot{V}}_D^\lambda } = \sum \limits _{k=1}^L \lambda _k^2 {{\dot{V}}_D^k} + \sum \limits _{k=1}^{L-1} \sum \limits _{j = k+1}^L \lambda _k \lambda _j \left( dV_D^{kj} + dV_D^{jk}\right) \end{aligned}$$

Using (34) and the definition of $\delta f_D^{kj}$, (35) can be written as:

36 $$\begin{aligned} \begin{aligned} {{\dot{V}}_D^\lambda }&= \sum \limits _{k=1}^L \lambda _k^2 {{\dot{V}}_D^k} +\\&+ \sum \limits _{k=1}^{L-1} \sum \limits _{j = k+1}^L \lambda _k \lambda _j \left( {{\dot{V}}_D^k} + {{\dot{V}}_D^j} + \left( \nabla V_D^j - \nabla V_D^k \right) \delta f_D^{jk} \right) \end{aligned} \end{aligned}$$

It is easy to show that:

37 $$\begin{aligned} \left( \nabla V_D^j - \nabla V_D^k \right) \delta f_D^{jk} = {\bar{x}}^T \delta {\tilde{P}}_D^{kj} {\bar{x}} \end{aligned}$$

with $\delta {\tilde{P}}_D^{kj}$ defined by (30), and that:

38 $$\begin{aligned} \sum \limits _{k=1}^{L-1} \sum \limits _{j = k+1}^L \lambda _k \lambda _j \left( {{\dot{V}}_D^k} + {{\dot{V}}_D^j}\right) = \sum \limits _{k=1}^{L} \lambda _k {{\dot{V}}_D^k} \sum \limits _{\begin{array}{c} j = 1 \\ j\ne k \end{array}}^L \lambda _l \end{aligned}$$

Given that $\lambda \in S_L$, it holds:

$$\begin{aligned} \sum \limits _{\begin{array}{c} j = 1 \\ j\ne k \end{array}}^L \lambda _l = 1 - \lambda _k \end{aligned}$$

and so using (37) and (38), Eq. (35) can be written as:

39 $$\begin{aligned} {{\dot{V}}_D^\lambda } = \sum \limits _{k=1}^L \lambda _k {{\dot{V}}_D^k} + \sum \limits _{k=1}^{L-1} \sum \limits _{j = k+1}^L \lambda _k \lambda _j {\bar{x}}^T \delta {\tilde{P}}_D^{kj} {\bar{x}} \end{aligned}$$

which, given the constraints (29), immediately gives the following:

40 $$\begin{aligned} {{\dot{V}}_D^\lambda }(x) \le \max \limits _{k \in \{1, \ldots L\}}\{{{\dot{V}}_D^k}(x)\} \end{aligned}$$

At this point using the fact that any extremal Lyapunov function $V^k$ satisfies (12), it follows that also $V^\lambda$ satisfies (12), proving that $V^\lambda$ is a PWQ-LF for $\sigma ^\lambda$. The same reasoning is valid for any $\lambda \in S_L$, and this completes the proof. $\square$

#### Remark 6

We refer to the set of LMIs (29) as crossed conditions, as each one of these connects different extremal Lyapunov functions and different extremal systems, inside the same regulatory domain. The need for these conditions is made explicit in equation (39), as the derivative in time of the Lyapunov function $V_D^\lambda$ is not only dependent on the derivatives in time of the extremal Lyapunov functions, but also on a set of crossed terms, which are guaranteed to be non-positive if the crossed conditions are satisfied.

These terms are a consequence of the fact that both the system $\sigma ^\lambda$ and the chosen PD-LF $V^\lambda$ are convex combinations of their extremal counterparts. $\blacktriangleleft$

### Extended feasibility problem definition

In the previous Sections we introduced additional LMIs to deal with monotonicity of the PD-LF in switching and regulatory domains. Now it is possible to redefine the Feasibility Problem in Pasquini and Angel (2019) [i.e. the set of LMIs that enforce the constraints (12)–(14)] to include these modified conditions. Given the notation and discussions from the previous sections, we aim to solve the following extended feasibility problem.

#### Problem 1

(*Extended feasibility problem*) Let $\varSigma _1, \ldots , \varSigma _L$ be *L* extremal systems as defined in (16), and consider the associated domain partition, as described in Sect. 3. Find *L* Piecewise Quadratic (PWQ) functions $V^k(x)$:

$$\begin{aligned} V^k(x) = V^k_D(x) = x^T P^k_D x + 2 {d_D^k}^T x + \omega ^k_D, \qquad x \in D,\ D \in {\mathscr {D}}_R \end{aligned}$$

subject to the following constraints:

41 $$\begin{aligned} \begin{array}{lll}&\varGamma _D^T {\tilde{P}}^k_D \varGamma _D + M^k_D \preceq 0,&\quad \forall D \in {\mathscr {D}}_R, \ \forall k \in \{1,\ldots ,L\} \end{array} \end{aligned}$$

42 $$\begin{aligned} \begin{array}{lll} &{}\varGamma _{D_s}^T \big ({\overline{P}}^k_D - {\overline{P}}^k_{D'}\big ) \varGamma _{D_s} = 0, &{}\quad \forall D, D' \in R(D_s),\ \forall k \in \{1,\ldots ,L\} \\ &{}&{}\quad \forall D_S \text { s.t } {\mathscr {P}}_{D_s} \ne \emptyset \end{array} \end{aligned}$$

43 $$\begin{aligned} \begin{array}{lll} &{}\varGamma _{D_s}^T L^k_{D_s,i} \varGamma _{D_s} + M^k_{D_s} \preceq 0, &{}\quad \forall i \in \{1, \ldots , \nu \},\ \forall k \in \{1,\ldots ,L\} \\ &{}&{}\quad \forall D_S \text { s.t } {\mathscr {P}}_{D_s} \ne \emptyset \\ {\mathscr {P}}_{D_s} = \text {conv}(W), &{} W := \{w_1,\ldots ,w_{\nu _\gamma }\} \end{array} \end{aligned}$$

44 $$\begin{aligned} \begin{array}{lll} &{}\varGamma _{D_\emptyset }^T ({\overline{P}}^k_{D_1} - {\overline{P}}^k_{D_2}) \varGamma _{D_\emptyset } = 0, &{}\quad \forall D_1, D_2 \in {\bar{R}}(D_i) \cup {\bar{R}}(D_j), \ \forall k \in \{1,\ldots ,L\} \\ &{}&{}\quad \forall \{N_{D_i} \rightarrow N_{D_\emptyset } \rightarrow N_{D_j}\} \in C \\ &{}&{}\quad \forall C \in {\mathscr {C}} \end{array} \end{aligned}$$

45 $$\begin{aligned} \begin{array}{lll} &{}\varGamma _D^T \delta {\tilde{P}}_D^{kj} \varGamma _D + M_D^{kj} \preceq 0, &{}\forall D \in {\mathscr {D}}_R, \forall k \in \{1,\ldots ,L-1\} \\ &{}&{}\forall j \in \{k+1,\ldots ,L\} \end{array} \end{aligned}$$

$$\blacktriangleleft $$

Solving Problem 1 (if feasible) will give *L* PWQ Lyapunov functions, that when combined as in (32), return a function $V^\lambda$ that will be referred as a Parameter Dependent PWQ Lyapunov function for the systems in ${\mathscr {U}}_1^L$.

#### Remark 7

Constraints (42) and (44) are connected to the continuity of extremal LFs in switching domains with sliding modes and along STG cycles. $\blacktriangleleft$

#### Remark 8

The decision variables in Problem 1 are the components of the extremal Lyapunov functions: $P^k_D$, $d^k_D$ and $\omega ^k_D$, for each regulatory domain *D*, together with the entrywise non-negative matrices *M*s [in constraints (41), (45) and (43)]. Numerical optimization LMI solvers are used to determine a valid solution of this problem e.g. the YALMIP interface (Löfberg 2004) in MATLAB, with the SDPT3 solver. $\blacktriangleleft$

#### Remark 9

The main impact in terms of computational complexity, is connected to switching domains, despite the fact that a switching domain $D_s$ enters in Problem 1 only if the associated polyhedron ${\mathscr {P}}_{D_s}$, described in (21), is non-empty. In particular the number of switching domains increase exponentially with the size of the network and the number of thresholds, and while the operation of checking whether a polyhedron is empty or not can be performed efficiently, transform a non-empty polyhedron from its *H*-representation to its *V*-representation, as it is needed in Problem 1, is a non-polynomial operation. We recognise this to be a potential drawback of the approach, to be addressed in further research. $\blacktriangleleft$

#### Remark 10

It is reasonable to wonder if results on Lyapunov functions that are valid for hybrid systems transfer, in some way, to the case where the regulation function is smoothly close to its discontinuous counterpart (i.e. when its Hill coefficient is large enough).

Lyapunov functions with strictly negative derivative are robust to sufficiently small perturbations of the dynamics (e.g. by considering it $C^0$ close to PWA). Despite in our case we do not ask for strict negativity of the derivative, converse Lyapunov results, for differential inclusions (see Forni and Angeli 2017) guarantee existence of Lyapunov functions with strictly negative derivative and therefore affording some kind of robustness with respect to these perturbations. The implications of this are not explored in this context, but are certainly of interest for future research. $\blacktriangleleft$

### The set-valued derivative map

We recall and adapt the following definition from Pasquini and Angel (2019).

#### Definition 1

Let $V^k$ be a PWQ-LF for the extremal system $\varSigma ^k$ as defined in (16), obtained as a solution of Problem 1. Let $x \in D$. The set-valued map $\overset{ \circ \,k}{V}(x)$ is defined as:

46 $$\begin{aligned} \overset{ \circ \,k}{V}(x) := \nabla V^k_D(x) \cdot \big (f^k_D - C x\big ) \end{aligned}$$

if $$D \in {\mathscr {D}}_R$$, or:

47 $$\begin{aligned} \overset{ \circ \,k}{V}(x) := {\left\{ \begin{array}{ll} \begin{array}{lr} \emptyset \ &{}\quad \text {if } {\mathscr {P}}_{D} = \emptyset \\ \{\overline{\nabla V^k}_{{\hat{D}}}(x) \cdot (F w_i - C x), w_i \in W\} &{}\quad \text {if } {\mathscr {P}}_{D} \ne \emptyset \end{array} \end{array}\right. } \end{aligned}$$

if $D \in {\mathscr {D}}_S$, with ${\hat{D}}$ being any regulatory domain in *R*(*D*), $\overline{\nabla V^k}_{{\hat{D}}}(x)$ defined as ${\nabla V^k}_{{\hat{D}}}$ on the closure of ${\hat{D}}$, ${\mathscr {P}}_{D}$ being the polyhedron (21) for *D*, *F* being defined as in (27) and *W* being the vertices set of ${\mathscr {P}}_{D}$’s V-representation [as expressed in (24)]. $\blacktriangleleft$

The set-valued map $\overset{\circ }{V}^k$ is connected to the derivative of $V^k$ along the system trajectories, through the following Lemma, proved in Pasquini and Angel (2019).

#### Lemma 1

Consider a PWQ-LF $V^k(x)$ for system (16). Let ${\tilde{x}}(\cdot )$ be a solution of (16) on the interval *I*. Then ${d \over dt}V^k({\tilde{x}}(t))$ is a convex combination of the elements in the set $\overset{\circ }{V}^k({\tilde{x}}(t))$ for a.e. $t \in I$.

When we consider a system $\sigma ^\lambda \in C^1_L$ with polytopic uncertainties, Definition 1 could be naturally extended to the following:

#### Definition 2

Let $\sigma ^\lambda \in {\mathscr {U}}_1^L$, with ${\mathscr {U}}_1^L$ defined as in (17), let $V^1, \ldots , V^L$ be extremal LFs for the extremal systems $\varSigma _1, \ldots , \varSigma _L$ (obtained as solutions of Problem 1) and let $V^\lambda$ be the PD-PWQ-LF for $\sigma ^\lambda$ defined in (32). Let $x \in D$. The set-valued map $\overset{ \circ \,\lambda }{V}(x)$ is defined as:

48 $$\begin{aligned} \overset{ \circ \,\lambda }{V}(x) := \nabla V^\lambda _D(x) \cdot (f^\lambda _D - C x) \end{aligned}$$

if $$D \in {\mathscr {D}}_R$$, where

49 $$\begin{aligned} \begin{array}{rl} \nabla V^\lambda _{D}(x) &{}= \sum \limits _{k = 1}^L \lambda _k \nabla V^k_{D}(x)\\ f_D^\lambda &{}= \sum \limits _{k = 1}^L \lambda _k f_D^k \end{array} \end{aligned}$$

or:

50 $$\begin{aligned} \overset{ \circ \,\lambda }{V}(x) := {\left\{ \begin{array}{ll} \begin{array}{ll} \emptyset \ &{}\quad \text {if } {\mathscr {P}}_{D} = \emptyset \\ \{\overline{\nabla V^\lambda }_{{\hat{D}}}(x) \cdot (F w_i - C x), w_i \in W\} &{}\quad \text {if } {\mathscr {P}}_{D} \ne \emptyset \end{array} \end{array}\right. } \end{aligned}$$

if $D \in {\mathscr {D}}_S$, with ${\hat{D}}$ being any regulatory domain in *R*(*D*), $\overline{\nabla V^\lambda }_{{\hat{D}}}(x)$ be defined as ${\nabla V^\lambda }_{{\hat{D}}}$ on the closure of ${\hat{D}}$, ${\mathscr {P}}_{D}$ being the polyhedron (21) for *D*, *F* being defined as in (27) and *W* being the vertices set of ${\mathscr {P}}_{D}$’s V-representation [as expressed in (24)]. $\blacktriangleleft$

#### Remark 11

The definition of $\overset{ \circ \,\lambda }{V}$ on switching domains, takes into account the possible sliding mode directions of all the extremal systems, being ${\mathscr {P}}_{D}$ defined as in (21). This choice is conservative, but it is legitimate as it surely contains all directions of interest, and seems necessary due to the fact that the set of sliding mode directions it is not easily described in terms of $\lambda$. Moreover we remark that for regulatory domains the set-valued map $\overset{\circ }{V}$ and the function ${\dot{V}}$ are equivalent.$\blacktriangleleft$

To complete this section we give a result about some bounds on the elements of the map $\overset{\circ }{V}$, which will be useful in proving subsequent results.

#### Corollary 1

Let $\varSigma _1$, $\ldots$, $\varSigma _L$ be *L* extremal systems as defined in (16) and let $V^1$, $\ldots$, $V^L$ be their extremal LFs. Let the conditions of Theorem 1 be satisfied and let $V^\lambda$ be the PD-PWQ-LF defined in (19). Let $D \in {\mathscr {D}}_R$. Then:

51 $$\begin{aligned} \overset{ \circ \,\lambda }{V}(x) \le \max \limits _{k \in \{1, \ldots L\}}\left\{ \overset{ \circ \,k}{V}(x)\right\} , \quad x \in D \end{aligned}$$

#### Proof

(51) follows from (40) and the fact that ${\dot{V}}$ and $\overset{\circ }{V}$ are equivalent in regulatory domains. $\square$

### Robust convergence properties

It is easy to extend Proposition 1 to prove that a condition like (15) holds for any system $\sigma ^\lambda \in {\mathscr {U}}_1^L$, i.e.:

52 $$\begin{aligned} \lim \limits _{\tau \rightarrow \infty } \mu \left( \left\{ t \ge \tau : {d V^\lambda (x(t)) \over d t} < -\varepsilon \right\} \right) = 0 , \qquad \forall \varepsilon > 0, \quad \forall \lambda \in S_L \end{aligned}$$

However we would like to give results which take into account all the possible realizations of the uncertain system, to state convergence properties which are robust to the polytopic uncertainty.

With respect to the set-valued maps $\overset{\circ }{V}$ we introduce the following set:

53 $$\begin{aligned} \varOmega ^+(\overset{\circ }{V},\varepsilon ) = \{x \in {\mathbb {R}}^n_+ \ |\ \max \{\overset{\circ }{V}(x)\} \ge -\varepsilon \} \end{aligned}$$

It is remarked that the set $\varOmega ^+$ can be evaluated for both $\overset{ \circ \,\lambda }{V}$ and $\overset{ \circ \,k}{V}$. Consider a $\lambda \in S_L$ and let $\overset{ \circ \,\lambda }{V}$ and $\varOmega ^+$ be defined as in Definition 2 and Eq. (53) respectively. The following intuitive Proposition holds:

#### Proposition 2

Let $x(\cdot )$ be a trajectory of system $\sigma ^\lambda$, let $V^\lambda$ be a PWQ Lyapunov function for the system, intended as a solution to Problem 1 and let $\overset{ \circ \,\lambda }{V}$ be the set-valued map defined in Definition 2. Let $\varOmega ^+(\overset{ \circ \,\lambda }{V},\varepsilon )$ be defined as in (53). Consider an interval $(t_0,t_1)$, with $t_0 < t_1$ ($t_1$ could be $\infty$). Then:

$$\begin{aligned} {dV(x(t)) \over dt} \ge -\varepsilon , \ \text { for a.e. } t \in (t_0, t_1) \ \ \Rightarrow \ \ x(t) \in \varOmega ^+(\overset{\circ }{V},\varepsilon ), \ \text { for a.e. } t \in (t_0, t_1) \end{aligned}$$

#### Proof

By Lemma 1, we know that ${dV^\lambda (x(t)) \over dt} \in \text {conv}\{\overset{ \circ \,\lambda }{V}(x(t))\}$ for a.e. $t \in (t_0, t_1)$ (and more in general, for a.e. *t* in the whole interval of definition of the solution $x(\cdot )$). Let ${dV^\lambda (x(t)) \over dt} \ge -\varepsilon$ and assume, by contradiction, that $\max \{\overset{ \circ \,\lambda }{V}(x(t))\} < -\varepsilon$ (i.e. $x(t) \notin \varOmega ^+(\overset{ \circ \,\lambda }{V},\varepsilon )$). Then any element in $\text {conv}\{\overset{ \circ \,\lambda }{V}(x(t))\}$ would be smaller than $-\varepsilon$, contradicting the fact that ${dV^\lambda (x(t)) \over dt} \ge -\varepsilon$. $\square$

For $\varepsilon \rightarrow 0$, the set $\varOmega ^+(\overset{ \circ \,\lambda }{V},\varepsilon )$ gives information on where the system will converge asymptotically, being in particular an outer approximation of the convergence set, and for this reason its study is of paramount importance. The same set can be defined also for the extremal Lyapunov function $V^k$, and will be denoted with $\varOmega ^+(\overset{ \circ \,k}{V},\varepsilon )$. Our goal is to characterize the set $\varOmega ^+(\overset{ \circ \,\lambda }{V},\varepsilon )$, as $\lambda$ varies in $S_L$, in order to guarantee robust convergence result. We are interested in a qualitative analysis, therefore the following definitions are justified.

#### Definition 3

Consider the extremal system $\varSigma ^k$ and its extremal Lyapunov function $V^k$. We call ${\overline{D}}^k_\varepsilon$ the set:

$$\begin{aligned} {\overline{D}}^k_\varepsilon := \big \{D \in {\mathscr {D}} \ | \ \exists x \in D \text { s.t. } \max \big \{\overset{ \circ \,k}{V}(x)\big \} \ge -\varepsilon \big \} \end{aligned}$$

with $${\mathscr {D}} = {\mathscr {D}}_R \cup {\mathscr {D}}_S$$. $$\blacktriangleleft $$

Namely ${\overline{D}}^k_\varepsilon$ is the set of domains containing $\varOmega ^+(\overset{ \circ \,k}{V},\varepsilon )$.

#### Definition 4

We call ${\overline{D}}_\varepsilon$ the union of the sets ${\overline{D}}^k_\varepsilon$ for all the extremal systems, formally:

$$\begin{aligned} {\overline{D}}_\varepsilon := \bigcup \limits _{k = 1}^L {\overline{D}}^k_\varepsilon \end{aligned}$$

$$\blacktriangleleft $$

#### Definition 5

Consider a system $\sigma ^\lambda \in {\mathscr {U}}_1^L$ admitting a PD-PWQ-LF $V^\lambda$. With analogy to Definition 3, we call ${\overline{D}}^\lambda _\varepsilon$ the set:

$$\begin{aligned} {\overline{D}}^\lambda _\varepsilon := \big \{D \in {\mathscr {D}} \ | \ \exists x \in D \text { s.t. } \max \big \{\overset{ \circ \,\lambda }{V}(x)\big \} \ge -\varepsilon \big \} \end{aligned}$$

$$\blacktriangleleft $$

As aformentioned, ${\overline{D}}^\lambda _\varepsilon$ is the set of domains containing at least a point of $\varOmega ^+(\overset{ \circ \,\lambda }{V},\varepsilon )$. Given that the dependence of $\varOmega ^+(\overset{ \circ \,\lambda }{V},\varepsilon )$ on $\lambda \in S_L$ is extremely hard to describe, we aim to define a relation in terms of domains (i.e. the relationship between ${\overline{D}}^k_\varepsilon$, ${\overline{D}}_\varepsilon$ and ${\overline{D}}^\lambda _\varepsilon$). The following Theorem gives such relationship.

#### Theorem 2

Let $\varSigma _1$, $\ldots$, $\varSigma _L$ be *L* extremal systems as defined in (16) and let $V^1$, $\ldots$, $V^L$ be their extremal LFs, obtained as solution of Problem 1. Let $V^\lambda$ be the PD-PWQ-LF defined in (32). Let ${\overline{D}}_\varepsilon ^\lambda$ and ${\overline{D}}_\varepsilon$ be defined as in Definition 4 and 5. Then:

$$\begin{aligned} {\overline{D}}^\lambda _\varepsilon \subseteq {\overline{D}}_\varepsilon , \qquad \forall \lambda \in S_L \end{aligned}$$

#### Proof

Let *D* be a switching domain, belonging to the set ${\overline{D}}_\varepsilon ^\lambda$. This implies that both ${\mathscr {P}}_D$ and $\overset{ \circ \,\lambda }{V}(x)$, for $x \in D$, are non-empty. Let ${\mathscr {P}}_D = \{w_1, \ldots , w_{\nu _\gamma }\}$, $\overset{ \circ \,\lambda }{V}(x)$ is expressed as:

54 $$\begin{aligned} \begin{aligned} \overset{ \circ \,\lambda }{V}(x) = \big \{ \overline{\nabla V}^\lambda _{{\hat{D}}}(x) \cdot (F w_1 - C x), \ \ldots , \ \overline{\nabla V}^\lambda _{{\hat{D}}}(x) \cdot (F w_{\nu _\gamma } - C x)\big \} \end{aligned} \end{aligned}$$

in which ${\hat{D}}$ is any regulatory domain adjacent to *D*. Being:

55 $$\begin{aligned} \overline{\nabla V}^\lambda _{{\hat{D}}}(x) = \sum \limits _{k = 1}^L \lambda _k \overline{\nabla V}^k_{{\hat{D}}}(x) \end{aligned}$$

let:

56 $$\begin{aligned} \xi ^{k,j}(x) := \overline{\nabla V}^k_{{\hat{D}}}(x) \cdot (F w_j - C x) \end{aligned}$$

Notice that $\xi ^{k,j}(x)$ is an element of $\overset{ \circ \,k}{V}(x)$, for any $x \in D$ and any $j \in \{1, \ldots , \nu _\gamma \}$. Since $D \in {\overline{D}}_\varepsilon ^\lambda$:

57 $$\begin{aligned} \exists x \in D \quad \text {s.t.} \quad \max \limits _{j \in \{1, \ldots \nu _\gamma \}}\left\{ \sum \limits _{k = 1}^L\lambda _k \xi ^{k,j}(x)\right\} \ge -\varepsilon \end{aligned}$$

Assume, for the sake of contradiction, that $\xi ^{k,j}(x) < -\varepsilon$ for every *k*, *j* and $x \in D$ (i.e. there is no *k* such that $D \in {\underline{D}}_\varepsilon ^k \subseteq {\underline{D}}_\varepsilon$). Because $\lambda \in S_L$, this yields to:

$$\begin{aligned} \max \limits _{j \in \{1, \ldots \nu _\gamma \}}\left\{ \sum \limits _{k = 1}^L\lambda _k \xi ^{k,j}(x)\right\} < -\varepsilon , \quad \forall x \in D \end{aligned}$$

which contradicts (57), proving that $D \in {\overline{D}}_\varepsilon$. We now turn to prove the same inclusion for regulatory domains.

Let *D* be a regulatory domain, belonging to the set ${\overline{D}}_\varepsilon ^\lambda$. Similarly to the previous case, it holds:

$$\begin{aligned} \exists x \in D \quad | \quad \overset{ \circ \,\lambda }{V}(x) \ge -\varepsilon \end{aligned}$$

From Corollary 1, it follows:

$$\begin{aligned} -\varepsilon \le \overset{ \circ \,\lambda }{V}(x) \le \max \limits _{k \in \{1, \ldots L\}} \big \{\overset{ \circ \,k}{V}(x)\big \} \end{aligned}$$

implying that $\exists k \in \{1, \ldots , L\}$, for which:

$$\begin{aligned} \overset{ \circ \,k}{V}(x) \ge -\varepsilon \end{aligned}$$

proving that $D \in {\overline{D}}_\varepsilon ^k \subseteq {\overline{D}}_\varepsilon$. This concludes the proof. $\square$

#### Corollary 2

Given the premises of Theorem 2, it holds:

$$\begin{aligned} {\overline{D}}_\varepsilon = \bigcup \limits _{\lambda \in S_L} {\overline{D}}^\lambda _\varepsilon \end{aligned}$$

#### Proof

The fact that:

$$\begin{aligned} {\overline{D}}_\varepsilon \subseteq \bigcup \limits _{\lambda \in S_L} {\overline{D}}^\lambda _\varepsilon \end{aligned}$$

follows from the fact that any set ${\overline{D}}^k_\varepsilon$ can be obtained by selecting a vertex of the symplex $S_L$. The fact that:

$$\begin{aligned} \bigcup \limits _{\lambda \in S_L} {\overline{D}}^\lambda _\varepsilon \subseteq {\overline{D}}_\varepsilon \end{aligned}$$

directly follows from Theorem 2. $\square$

As hinted above, the set ${\overline{D}}_\varepsilon$ contains information on the convergence set, for all the systems in ${\mathscr {U}}_1^L$, a fact that will be formalised by Proposition 3 below, but before proceeding to state the proposition and its proof we need to introduce the concept of sink regulatory domains. In particular some regulatory domains have the property that whenever a trajectory enter them, it doesn’t leave. Formally we call sink domain any regulatory domain *D*, for which $\varPhi (D) \in D$, where $\varPhi (D)$ is the focal point of *D*.

The analysis inside sink domains is trivial (Casey et al. 2006) and to include them in the feasibility problem to be solved does not add any information to the study of convergence. At the same time they restrict the set of feasible extremal Lyapunov functions that satisfy the constraints in Theorem 1, as $\varPhi (D)$ "moves" with the presence of uncertainties, as the ones described above, in a way that is nearly impossible to capture with a PD-PWQ-LF linear in the extremal LFs. On the other hand a bound on the position of $\varPhi (D)$ could be easily computed and we know that in *D* the trajectories will monotonically converge to $\varPhi (D)$.

For these reasons, we remove from the analysis any sink domain, including them in the convergence set, as a trajectory could always enter (or start in) a sink domain and converge to their focal point.

#### Proposition 3

Let $\varSigma _1$, $\ldots$, $\varSigma _L$ be *L* extremal systems as defined in (16) and let $V^1$, $\ldots$, $V^L$ be their extremal LFs, obtained as solution of Problem 1. Let $\lambda \in S_L$ and let $V^\lambda$ be the PD-PWQ-LF, as defined in (32), for $\sigma ^\lambda \in {\mathscr {U}}_1^L$. Let ${\overline{D}}_\varepsilon$ be the set in Definition 4 and let $\mathscr {SD}$ be the set of sink domains. Let $x(\cdot )$ be a solution of $\sigma ^\lambda$ and let *D*(*x*(*t*)) be the function that associates the point *x*(*t*) to the domain it belongs. Then:

58 $$\begin{aligned} \lim \limits _{\tau \rightarrow \infty } \mu (\{t \ge \tau \ | \ D(x(t)) \in {\mathscr {D}} {\setminus } \{{\overline{D}}_\varepsilon \cup \mathscr {SD}\}) = 0 , \qquad \forall \varepsilon > 0 \end{aligned}$$

#### Proof

From (52) we know that:

$$\begin{aligned} \lim \limits _{\tau \rightarrow \infty } \mu \left( \left\{ t \ge \tau : {d V^\lambda \over d t} < -\varepsilon \right\} \right) = 0 , \qquad \forall \varepsilon > 0 \end{aligned}$$

Let $\varOmega ^+(\overset{ \circ \,\lambda }{V},\varepsilon )$ be the set (53) for $V^\lambda$. It follows easily from Lemma 1 that ${dV^\lambda (x(t)) \over dt} \in \text {conv}\{\overset{ \circ \,\lambda }{V}\}$ for a.e. *t* and from Proposition 2, it follows that:

$$\begin{aligned} {dV^\lambda (x(t)) \over dt} \ge -\varepsilon \ \forall t \in [0,\infty ) {\setminus } {\mathscr {T}} \Rightarrow x(t) \in \varOmega ^+\big (\overset{ \circ \,\lambda }{V},\varepsilon \big ) \ \ \forall t \in [0,\infty ) {\setminus } {\mathscr {T}} \end{aligned}$$

where ${\mathscr {T}}$ is a set of times whose measure is finite.

By definition of ${\overline{D}}_\varepsilon$ and Theorem 2, it holds:

$$\begin{aligned} x(t) \in \varOmega ^+\big (\overset{ \circ \,\lambda }{V},\varepsilon \big ) \Rightarrow D(x(t)) \in {\overline{D}}_\varepsilon \end{aligned}$$

The fact that $V^\lambda (t)$ is absolutely continuous (being the convex combination of absolutely continuous functions—see Pasquini and Angel (2019) implies that the derivative $dV^\lambda \over dt$ exists almost everywhere. This fact and the property that any sink domain is positively invariant, prove the thesis. $\square$

#### Remark 12

In the proof of Proposition 3 we used the fact that if $A \subseteq [0,\infty )$ then:

$$\begin{aligned} \mu (A) < \infty \iff \lim \limits _{\tau \rightarrow \infty } \mu (A \cap [\tau ,\infty )) = 0 \end{aligned}$$

where $\mu$ is the Lebesgue measure of the set *A*. $\blacktriangleleft$

#### Remark 13

Proposition 3 states that the trajectories will spend, asymptotically, most of the time in the domains in ${\overline{D}}_\varepsilon \cup \mathscr {SD}$, in the sense that, given a trajectory $x(\cdot )$, the measure of the set of times spent away from this set, shrinks to 0 as $t \rightarrow \infty$. $\blacktriangleleft$

#### Remark 14

In this work we considered polytopic uncertainties on the production rates only. However the overall analysis and LMIs framework can be easily adapted to handle uncertainties on the degradation rates only, changing the structure of the matrix $\delta {\tilde{P}}_D^{kj}$ in (30), or on both the production and degradation rates, by considering all the possible combinations of their extremal values, as vertices of the uncertainty polytope. $\blacktriangleleft$

## Numerical example

**Fig. 6:**
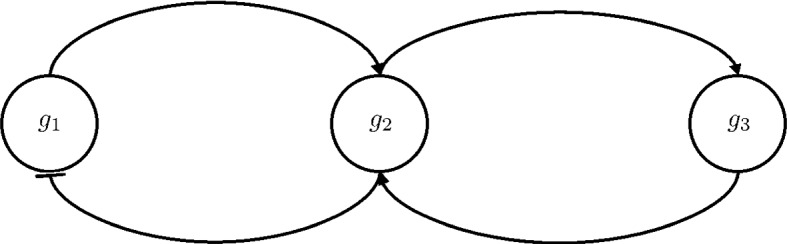
Genetic regulatory network of a double feedback system. The system consists of two feedback loops—a positive and a negative one—connected together

Consider the GRN of a two connected feedback loops as in Fig. 6, and consider the following dynamics for the network:

59 $$\begin{aligned} {\left\{ \begin{array}{ll} {\dot{x}}_1 = b_1 s^-(x_2, \theta _2) - c_1 x_1\\ {\dot{x}}_2 = b_2 [s^+(x_3,\theta _3) + s^+(x_1,\theta _1)] - c_2 x_2 \\ {\dot{x}}_3 = b_3 s^+(x_2,\theta _2) - c_3 x_3 \end{array}\right. } \end{aligned}$$

where $$b_1 = 3$$, $$b_2 \in [1.3, 1.8]$$, $$b_3 \in [2, 6]$$, $$c_1 = c_3 = 1$$, $$c_2 = 2$$ and $$\theta _1 = \theta _2 = \theta _3 = 1$$.

**Fig. 7:**
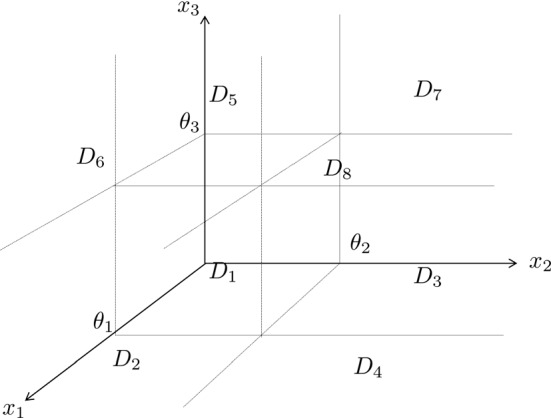
Box partition of the positive orthant for system (59)

System (59) has polytopic uncertainties on the parameters $b_2$ and $b_3$, so there are four extremal systems, corresponding to the possible combinations of $b_2$ and $b_3$ extrema.

Problem 1 is set up and hence the following constraints will be asked:

1. Monotonicity of every extremal Lyapunov function inside regulatory domains, together with the crossed conditions connecting couples of extremal Lyapunov functions and extremal systems i.e. constraints (41) and (45);
2. Monotonicity—and continuity—of every extremal Lyapunov function, in switching domains with non-empty ${\mathscr {P}}_D$ i.e. constraints (42) and (43). In this example, these domains are:
  $$\begin{aligned} \begin{aligned} D_{s_1}&= [0, \theta _1) \times \{\theta _2\} \times \{\theta _3\}\\ D_{s_2}&= \{\theta _1\} \times \{\theta _2\} \times (\theta _3, \infty )\\ \end{aligned} \end{aligned}$$
3. Continuity of the extremal Lyapunov functions along the cycles in the STG of the network, which converts into asking continuity on the walls connecting the domains $D_5$, $D_6$, $D_7$ and $D_8$, relatively to the partition in Fig. 7, as per constraints (44).

We can notice that the regulatory domain:

$$\begin{aligned} D_2 = [0,\theta _1) \times (\theta _2,\infty ) \times (\theta _3, \infty ) \end{aligned}$$

is a sink domain, and for this reason it is removed from the formulation of Problem 1.

**Fig. 8:**
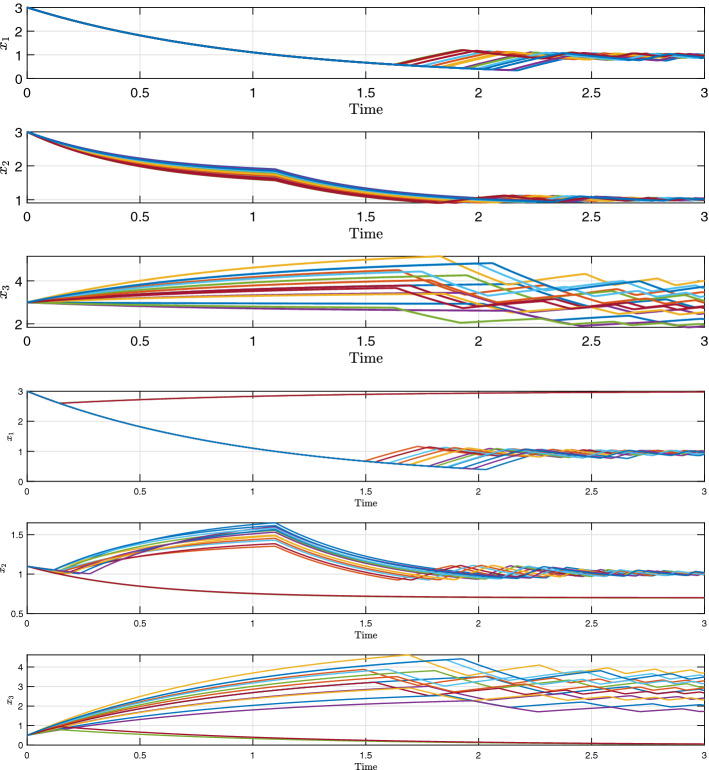
Trajectories of (59), for different realizations of the uncertainties on $b_2$ and $b_3$. Initial conditions are $[ 3 \ \ 3 \ \ 3]^T$ and $[3 \ \ 1.1 \ \ 0.5]^T$

**Fig. 9:**
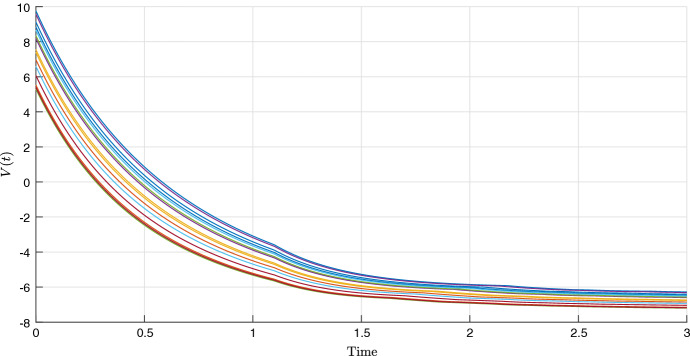
Evolution of the PD-LF $V^\lambda$, of (59), for different realizations of the uncertainties on $b_2$ and $b_3$. The initial condition is $[ 3 \ \ 3 \ \ 3]^T$ (color figure online)

**Fig. 10:**
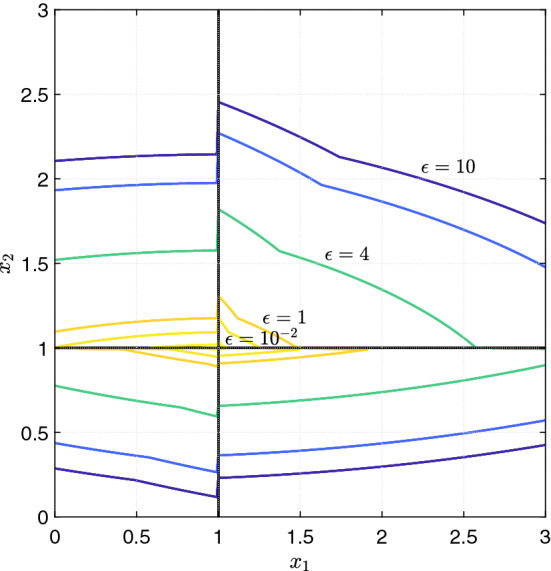
Level sets of the derivative map for system (59) (purple: $\varepsilon = 10$, yellow: $\varepsilon = 10^{-2}$) (color figure online)

**Fig. 11:**
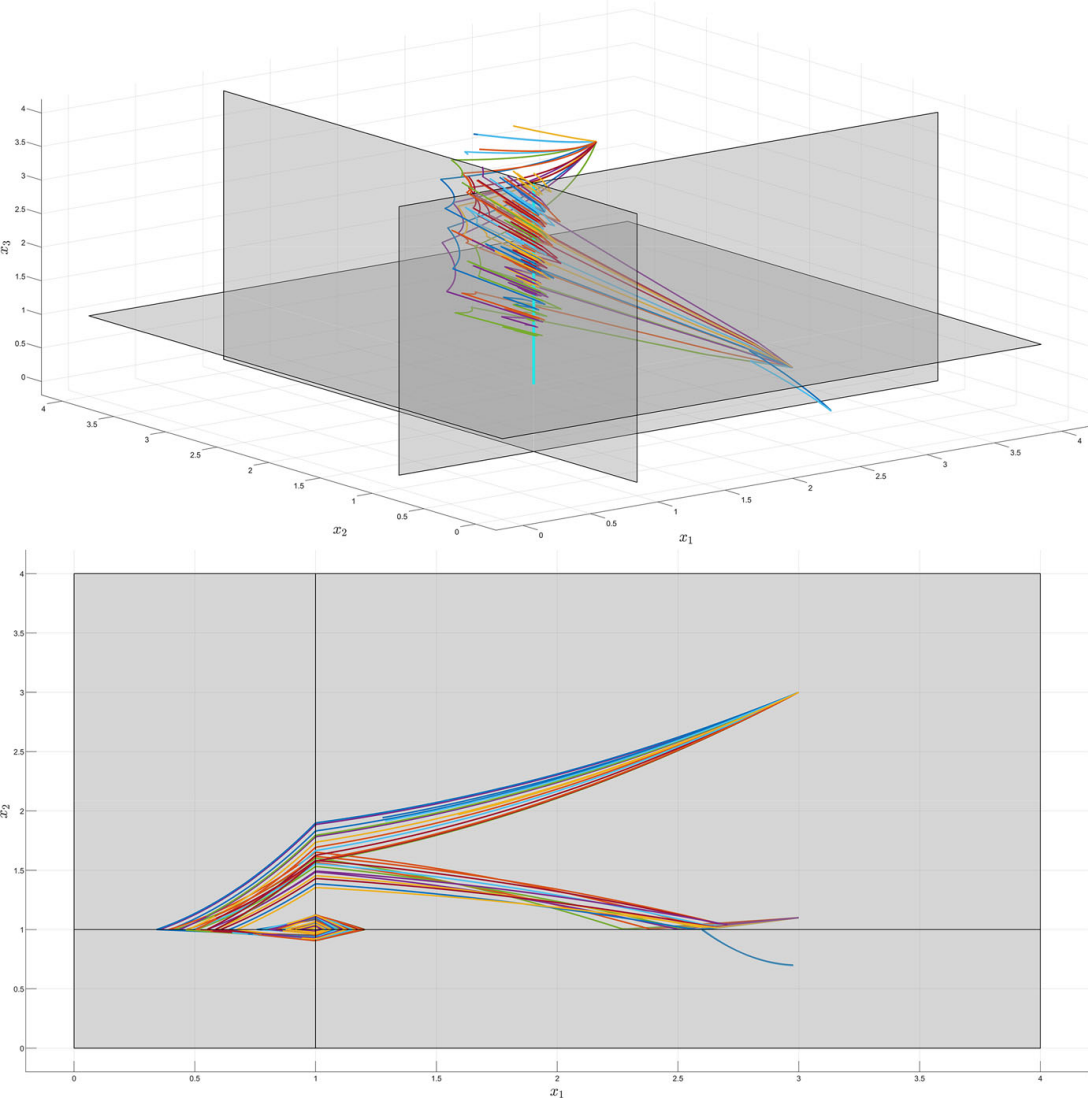
Phase space trajectories of (59), for different realizations of the uncertainties on $b_2$ and $b_3$—3d view and projection on $(x_1,x_2)$. The highlighted (cyan) domain is $D_{s_2}$, to which most of the trajectories converge, as predicted by our analysis (color figure online)

By solving Problem 1, it is possible to find four extremal Lyapunov functions, from which we can construct a PD-LF $V^\lambda$. In Fig. 8 some trajectories are shown for different realizations of the uncertainty and in Fig. 9 it is shown how the parameter dependent Lyapunov function $V^\lambda$, is eventually non-increasing along these trajectories. More numerical details on the LMI formulation and potential solution are given in the “Appendix”

Choosing $\varepsilon = 10^{-7}$, it is possible to find the sets ${\overline{D}}_\varepsilon ^k$, for all the extremal Lyapunov functions $V^k$, and consequently we can determine the set ${\overline{D}}_\varepsilon$, corresponding to:

$$\begin{aligned} {\overline{D}}_\varepsilon = \{D_{s_2}, D_5, D_6, D_7, D_8\} \end{aligned}$$

where:

$$\begin{aligned} \begin{aligned} D_{s_2}&= \{\theta _1\} \times \{\theta _2\} \times (\theta _3, \infty )\\ D_5&= [0,\theta _1) \times [0,\theta _2) \times (\theta _3,\infty )\\ D_6&= (\theta _1,\infty ) \times [0,\theta _2) \times (\theta _3,\infty )\\ D_7&= [0,\theta _1) \times (\theta _2,\infty ) \times (\theta _3,\infty )\\ D_8&= (\theta _1,\infty ) \times (\theta _2,\infty ) \times (\theta _3,\infty ) \end{aligned} \end{aligned}$$

Given the cyclic nature of the system trajectories around $D_{s_2}$, and because $\overset{ \circ \,k}{V}(x)$, for each extremal LF, approach 0 as *x* tends to $D_{s_2}$, the set ${\overline{D}}_\varepsilon$ contains also the regulatory domains adjacent to $D_{s_2}$ (i.e. $\{D_5, \ D_6, \ D_7, \ D_8\}$).

However from the proof of Theorem 2, it follows:

$$\begin{aligned} \overset{ \circ \,\lambda }{V}(x) \le \max \limits _{k \in \{1, \ldots L\}} \big \{\overset{ \circ \,k}{V}(x)\big \} \end{aligned}$$

so that, by studying the set where:

$$\begin{aligned} \max \limits _{k \in \{1, \ldots L\}} \big \{\overset{ \circ \,k}{V}(x)\big \} < -\varepsilon \end{aligned}$$

we can infer that the solutions always converge, in the sense of measure, to the domain $D_{s_2}$. This is also a consequence of the fact that $\overset{ \circ \,k}{V}$ is described, in the regulatory domains around $D_{s_2}$, by non-singular quadratic forms, and it is empty in the boundaries connecting these regulatory domains. To give a visual idea of the result, in Fig. 10, different level sets of the function $\max \limits _{k \in \{1, \ldots L\}} \{\overset{ \circ \,k}{V}(x)\}$ in the region $x_3 > \theta _3$ are shown, with decreasing values of $\varepsilon$. The projection on $(x_1,x_2)$ is considered, as $V^\lambda$ does not depend on $x_3$ in those particular regulatory domains.

This analysis is supported by the trajectories represented in Fig. 11, where the highlighted cyan domain is $D_{s_2}$, to which we can see that most of the trajectories converge. It should be noticed that, for the same initial condition $$x_0 = \begin{bmatrix} 3&1.1&0.5\end{bmatrix}^T$$, the system may converge to the switching domain $D_{s_2}$ or to the sink domain $D_2$, depending on the uncertainty realization.

### Remark 15

In Casey et al. (2006), a conjecture—proved true in Wang and Wang (2013) – is given with sufficient conditions for the weak asymptotic stability of a switching domain $D_s$, under the assumption that no cycles are present in the STG reduced to the neighbours of such switching domain. In the case of the above example however, the domain $D_{s_2}$ has two cycles involving neighbour domains, hence the result of Casey et al. (2006) and Wang and Wang (2013) cannot be applied. $\blacktriangleleft$

## Conclusion

Due to context-dependence and measurement limitations, models of biological systems are affected by parameter uncertainties and different kind of disturbances. In this chapter we considered a PWA model of genetic regulatory networks, and assumed the presence of polytopic uncertainties affecting the production rates. The LMI framework of Pasquini and Angel (2019) has been extended, allowing to determine a Parameter Dependent Piecewise Quadratic Lyapunov function, for the whole polytope of uncertain parameters. With this function we can conclude on the convergence properties of the trajectories to a particular set of domains, independently from the values assumed by the perturbed parameters. This can rule out a big chunk of the positive orthant from the convergence set, with further analysis that can be done in the resulting convergence set, to better characterise the system dynamics. In fact, as has been done in the double feedback example, the study of the level sets for the derivative map can allow to conclude more precise results on the system convergence.

These results can guide both in the synthesis of de-novo genetic circuits and in predicting the behavior of natural ones.

However there are a couple of issues in the analysis, that deserve to be addressed in future research. Firstly, as already addressed in Remark 3, Assumption 1 considers that the thresholds are unchanged among the extremal systems, which we recognize to be unrealistic. An extension of the framework, to allow polytopic uncertainties in the thresholds—e.g. by giving a lower and an upper-bound on each $\theta$—would certainly be of interest. However this would require a significant structural change of the framework, as continuity constraints would need to be applied on an undefined threshold, with their convexification leading to non-linear conditions, moving away from an LMI setup. If one chooses to drop any continuity constraint instead, the entire theoretical scaffolding should be changed, as infinite jumps of the Lyapunov functions would now be allowed, while at the same time, entry and exit points of sliding modes on the surface of discontinuity should be taken into account. These matters should be investigated in future research.

Secondly the constraints relative to the monotonicity of the Lyapunov function along sliding mode can be very conservative, and extremely expensive even to define. This is due to the fact that it is difficult to characterise the set of sliding directions in terms of the uncertain parameters, hence we need to impose this condition on all possible sliding directions, for all extremal systems. A better characterisation of this set—and the consequent relaxation of the LMI conditions—would increase the set of feasible solutions, while decreasing the computational cost of the approach.

In connection with this latter point, and Remark 9, general efficiency of the framework implementation should be pursued as many Genetic Regulatory Networks in nature are composed of tens of nodes. The framework described here can easily handle networks of up to six or seven nodes, but the computational cost becomes prohibitive after that. This work should be in fact considered as a proof of concept, and we believe that there are many possibilities to increase its efficiency.

Ultimately, future works should also consider control applications of the analytical results given above. Of particular interest is the problem of *in-vivo* control of Genetic Regulatory Networks, namely the addition, to the original GRN, of a second network whose goal is to obtain a desired overall behavior (e.g. control of an unstable steady state). With the current framework one could attempt a trial and error approach, meaning that a structure for the control-network can be chosen, the overall convergence properties studied through the described Lyapunov approach, and the control network modified accordingly, until the desired behavior is reached. We recognize that this approach is extremely inefficient and the possibility of embedding the control problem into a complementary set of LMIs should be addressed in future research.

## Appendix: Numerical details for the example of section 5

The positive orthant partition of system (59) gives rise to the following regulatory domains:

60 $$\begin{aligned} D_1&:= [0,\theta _1) \times [0,\theta _2) \times [0,\theta _3) \end{aligned}$$

61 $$\begin{aligned} D_2&:= (\theta _1,\infty ) \times [0,\theta _2) \times [0,\theta _3) \end{aligned}$$

62 $$\begin{aligned} D_3&:= [0,\theta _1) \times (\theta _2,\infty ) \times [0,\theta _3) \end{aligned}$$

63 $$\begin{aligned} D_4&:= (\theta _1,\infty ) \times (\theta _2,\infty ) \times [0,\theta _3) \end{aligned}$$

64 $$\begin{aligned} D_5&:= [0,\theta _1) \times [0,\theta _2) \times (\theta _3,\infty ) \end{aligned}$$

65 $$\begin{aligned} D_6&:= (\theta _1,\infty ) \times [0,\theta _2) \times (\theta _3,\infty ) \end{aligned}$$

66 $$\begin{aligned} D_7&:= [0,\theta _1) \times (\theta _2,\infty ) \times (\theta _3,\infty ) \end{aligned}$$

67 $$\begin{aligned} D_8&:= (\theta _1,\infty ) \times (\theta _2,\infty ) \times (\theta _3,\infty ) \end{aligned}$$

Given the uncertainty on $b_2$ and $b_3$, the following four extremal systems can be defined:

$$\begin{aligned} \varSigma _1&: {\left\{ \begin{array}{ll} {\dot{x}}_1 = b_1 s^-(x_2, \theta _2) - c_1 x_1\\ {\dot{x}}_2 = b_2^- [s^+(x_3,\theta _3) + s^+(x_1,\theta _1)] - c_2 x_2 \\ {\dot{x}}_3 = b_3^- s^+(x_2,\theta _2) - c_3 x_3 \end{array}\right. } \\ \varSigma _2&: {\left\{ \begin{array}{ll} {\dot{x}}_1 = b_1 s^-(x_2, \theta _2) - c_1 x_1\\ {\dot{x}}_2 = b_2^+ [s^+(x_3,\theta _3) + s^+(x_1,\theta _1)] - c_2 x_2 \\ {\dot{x}}_3 = b_3^- s^+(x_2,\theta _2) - c_3 x_3 \end{array}\right. } \\ \varSigma _3&: {\left\{ \begin{array}{ll} {\dot{x}}_1 = b_1 s^-(x_2, \theta _2) - c_1 x_1\\ {\dot{x}}_2 = b_2^- [s^+(x_3,\theta _3) + s^+(x_1,\theta _1)] - c_2 x_2 \\ {\dot{x}}_3 = b_3^+ s^+(x_2,\theta _2) - c_3 x_3 \end{array}\right. }\\ \varSigma _4&: {\left\{ \begin{array}{ll} {\dot{x}}_1 = b_1 s^-(x_2, \theta _2) - c_1 x_1\\ {\dot{x}}_2 = b_2^+ [s^+(x_3,\theta _3) + s^+(x_1,\theta _1)] - c_2 x_2 \\ {\dot{x}}_3 = b_3^+ s^+(x_2,\theta _2) - c_3 x_3 \end{array}\right. } \end{aligned}$$

where $$b_1 = 3$$, $$b_2^- = 1.3$$, $$b_2^+ = 1.8$$, $$b_3^- = 2$$, $$b_3^+ = 6$$, $$c_1 = c_3 = 1$$, $$c_2 = 2$$ and $$\theta _1 = \theta _2 = \theta _3 = 1$$.

Conditions (41) and (45), on the monotonicity inside regulatory domains and crossed conditions respectively, should be asked for each regulatory domain (with the only exception of the sink domain $D_2$) and each extremal Lyapunov function. Consider for example the domain $D_1$ and the first extremal Lyapunov function $V_1$. Condition (41) converts to:

68 $$\begin{aligned} \varGamma _{D_1}^T {\tilde{P}}^1_{D_1} \varGamma _{D_1} + M^1_{D_1} \preceq 0 \end{aligned}$$

where $\varGamma _{D_1}$ is the ray matrix of the homogenization cone ${\mathscr {C}}_{D_1}$, which can be constructed as in Eq. (3), in this case:

$$\begin{aligned} \varGamma _{D_1} = \begin{bmatrix} 0 &{}\quad \theta _1 &{}\quad 0 &{}\quad \theta _1 &{}\quad 0 &{}\quad \theta _1 &{}\quad 0 &{}\quad \theta _1 \\ 0&{}\quad 0&{}\quad \theta _2 &{}\quad \theta _2 &{}\quad 0 &{}\quad 0 &{}\quad \theta _2 &{}\quad \theta _2 \\ 0&{}\quad 0&{}\quad 0&{}\quad 0&{}\quad \theta _3&{}\quad \theta _3&{}\quad \theta _3&{} \quad \theta _3\\ 1&{}\quad 1&{}\quad 1&{}\quad 1&{}\quad 1&{}\quad 1&{}\quad 1&{}\quad 1 \end{bmatrix} \end{aligned}$$

while $${\tilde{P}}^1_{D_1}$$ is:

$$\begin{aligned} {\tilde{P}}^1_{D_1} = \begin{bmatrix} -2{\mathbf {P}}^{{\mathbf {1}}}_{{\mathbf {D}}_{\mathbf {1}}} C &{}\quad {\mathbf {P}}^{{\mathbf {1}}}_{{\mathbf {D}}_{\mathbf {1}}} f^1_{D_1} - C {\mathbf {d}}^{{\mathbf {1}}}_{{\mathbf {D}}_{\mathbf {1}}} \\ \big ({\mathbf {P}}^{{\mathbf {1}}}_{{\mathbf {D}}_{\mathbf {1}}} f^1_{D_1} - C {\mathbf {d}}^{{\mathbf {1}}}_{{\mathbf {D}}_{\mathbf {1}}}\big )^T &{}\quad 2 {\mathbf {d}}^{{\mathbf {1}}}_{{\mathbf {D}}_{\mathbf {1}}} f^1_{D_1} \end{bmatrix} \end{aligned}$$

for which ${\mathbf {P}}^{{\mathbf {1}}}_{{\mathbf {D}}_{\mathbf {1}}}$ and ${\mathbf {d}}^{{\mathbf {1}}}_{{\mathbf {D}}_{\mathbf {1}}}$ are decision variables, while:

$$\begin{aligned} C = \begin{bmatrix} 1 &{}\quad 0 &{}\quad 0\\ 0 &{}\quad 2 &{}\quad 0 \\ 0 &{}\quad 0 &{}\quad 1 \end{bmatrix}, \qquad f^1_{D_1} = \begin{bmatrix} b_1 \\ 0 \\ 0 \end{bmatrix} \end{aligned}$$

$M^1_{D_1}$ is another decision variable of the problem, with the only constraints that its elements are all non-negative. An LMI interpreter (such as Yalmip Löfberg 2004 in MATLAB) with a semidefinite programming solver (such as SDPT3 Toh et al. 1999) can be used to impose this kind of constraints and search for a solution. In regards to (45), consider for example the crossed conditions between the first and the second extremal Lyapunov functions (i.e. $V_1$ and $V_2$), for the regulatory domain $D_4$. This converts to:

69 $$\begin{aligned} \varGamma _{D_4}^T \delta {\tilde{P}}_{D_4}^{12} \varGamma _{D_4} + M_{D_4}^{12} \preceq 0, \qquad \end{aligned}$$

where:

$$\begin{aligned} \varGamma _{D_4} = \begin{bmatrix} \theta _1 &{}\quad \theta _1 &{}\quad 1 &{}\quad 0 \\ \theta _2 &{}\quad \theta _2 &{}\quad 0 &{}\quad 1 \\ 0 &{}\quad \theta _3 &{}\quad 0 &{}\quad 0 \\ 1 &{}\quad 1 &{}\quad 0 &{}\quad 0 \end{bmatrix} \end{aligned}$$

while:

$$\begin{aligned} \delta {\tilde{P}}_{D_4}^{12} = \begin{bmatrix} 0 &{}\quad -\big ({\mathbf {P}}^{\mathbf {2}}_{{\mathbf {D}}_{\mathbf {4}}} - {\mathbf {P}}^{\mathbf {1}}_{{\mathbf {D}}_{\mathbf {4}}}\big ) \big (f^2_{D_4} - f^1_{D_4}\big ) \\ -\big (f^2_{D_4} - f^1_{D_4}\big )^T \big ({\mathbf {P}}^{\mathbf {2}}_{{\mathbf {D}}_{\mathbf {4}}} - {\mathbf {P}}^{\mathbf {1}}_{{\mathbf {D}}_{\mathbf {4}}}\big ) &{}\quad -2 \big ({\mathbf {d}}_{{\mathbf {D}}_{\mathbf {4}}}^{\mathbf {2}} - {\mathbf {d}}_{{\mathbf {D}}_{\mathbf {4}}}^{\mathbf {1}}\big )^T \big (f^2_{D_4} - f^1_{D_4}\big ) \end{bmatrix} \end{aligned}$$

where, as previously, ${\mathbf {P}}^{\mathbf {2}}_{{\mathbf {D}}_{\mathbf {4}}}$, ${\mathbf {P}}^{\mathbf {1}}_{{\mathbf {D}}_{\mathbf {4}}}$, ${\mathbf {d}}^{\mathbf {1}}_{{\mathbf {D}}_{\mathbf {4}}}$, ${\mathbf {d}}^{\mathbf {2}}_{{\mathbf {D}}_{\mathbf {4}}}$ and $M_{D_4}^{12}$ are decision variables, while:

$$\begin{aligned} f^1_{D_4} = \begin{bmatrix} 0 \\ b_2^- \\ b_3^- \end{bmatrix} \qquad f^2_{D_4} = \begin{bmatrix} 0 \\ b_2^+ \\ b_3^- \end{bmatrix} \end{aligned}$$

We remark that conditions (68) and (69) are asked for all regulatory domains (except $D_2$) and all extremal Lyapunov functions (with the crossed conditions, asked for any possible couple of extremal Lyapunov functions).

For switching domains the first step is to check if, for any given $D_S$, the polyhedron ${\mathscr {P}}_{D_S}$ described in Eq. (21) is non-empty. This operation can be done efficiently using available software libraries for polyhedra (e.g. MPT3 Herceg et al. 2013). In this case the only two switching domains with such properties are:

$$\begin{aligned} \begin{aligned} D_{S_1}&= [0, \theta _1) \times \{\theta _2\} \times \{\theta _3\}\\ D_{S_2}&= \{\theta _1\} \times \{\theta _2\} \times (\theta _3, \infty )\\ \end{aligned} \end{aligned}$$

The *H*-representation (21) of the polyhedron $P_{D_S}$, is then converted to its *V* - representation, and again this operation can be performed using dedicated software toolboxes (e.g. MPT3). Condition (43) is then asked, for all vertices in such *V*-representation and all extremal systems. It is remarked that the polyhedron ${\mathscr {P}}_{D_S}$ is independent of the particular extremal Lyapunov function, by construction.

Moreover for the two switching domains, continuity of the extremal Lyapunov functions is asked. Consider for example the switching domain $D_{S_2}$ and the first extremal LF. Condition (42) converts to:

70 $$\begin{aligned} \varGamma _{D_{S_2}}^T \big (\overline{P^1_{D}}-\overline{P^1_{D'}}\big ) \varGamma _{D_{S_2}} = 0 \end{aligned}$$

where:

$$\begin{aligned} \varGamma _{D_{S_2}} = \begin{bmatrix} \theta _1 &{}\quad 0\\ \theta _2 &{}\quad 0\\ \theta _3 &{}\quad 1\\ 1 &{}\quad 0 \end{bmatrix} \end{aligned}$$

while $\overline{P^1_{D}}$ is the matrix:

$$\begin{aligned} \overline{P^1_{D}} = \begin{bmatrix} {\mathbf {P}}^{\mathbf {1}}_{\mathbf {D}} &{}\quad {\mathbf {d}}^{\mathbf {1}}_{\mathbf {D}} \\ {\mathbf {d}}^{\mathbf {1,T}}_{\mathbf {D}} &{}\quad \varvec{\omega }_{\mathbf {D}} \end{bmatrix} \end{aligned}$$

Condition (70) should be asked for all *D* and $D'$ belonging to $R(D_{S_2})$, i.e. the set of regulatory domains that have $D_{S_2}$ in their boundaries, in this case:

$$\begin{aligned} R(D_{S_2}) = \{D_5, D_6, D_7, D_8\} \end{aligned}$$

Condition (70) is then asked for all extremal Lyapunov functions and for all the other switching domains with non-empty ${\mathscr {P}}_{D_S}$, in this case only $D_{S_1}$.

Ultimately the conditions on continuity along cycles of the STG should be asked. Given the STG (which can be obtained through the algorithm explained in De Jong et al. 2004), we can search for all the cycles in it using efficient algorithms (see for example Tiernan 1970), and for each cycle ask for the extremal Lyapunov functions to be continuous on the switching domains which will eventually be crossed. In this case this converts to asking for the extremal Lyapunov function to be continuous on the walls connecting the regulatory domains $D_5$, $D_6$, $D_7$ and $D_8$. Consider for example the wall between $D_5$ and $D_6$, for the extremal Lyapunov function $V_1$. The continuity condition becomes:

$$\begin{aligned} \varGamma _{D_{56}}^T \big (\overline{P^1_{D_5}}-\overline{P^1_{D_6}}\big ) \varGamma _{D_{56}} = 0 \end{aligned}$$

where $D_{56}$ is the wall between $D_5$ and $D_6$, while:

$$\begin{aligned} \varGamma _{D_{56}} = \begin{bmatrix} \theta _1 &{}\quad \theta _1 &{}\quad 0 \\ 0 &{}\quad \theta _2 &{}\quad 0 \\ \theta _3 &{}\quad \theta _3 &{}\quad 1 \\ 1 &{}\quad 1 &{} \quad 0 \end{bmatrix} \end{aligned}$$

This type of condition is repeated for the remaining walls and for all other extremal Lyapunov functions.

Once all the LMIs and matrix equalities are collected, the feasibility problem can be solved (with the aforementioned software) and a description of the parameter dependent Lyapunov function (in terms of the variables $\mathbf {P^k_D}$, $\mathbf {d^k_D}$ and $\mathbf {\omega ^k_D}$ for all regulatory domains *D*, for all extremal Lyapunov functions $V^k$). As an example, below the description of the first extremal Lyapunov function:

$$\begin{aligned}&{\overline{P}}^1_{D_1} = \begin{bmatrix} 0.2126 &{}\quad 0 &{}\quad -\,0.3084 &{}\quad -\,0.7466 \\ 0 &{}\quad 0.3685 &{}\quad 0 &{}\quad 0.0913 \\ -\,0.3084 &{}\quad 0 &{}\quad 0.419 &{}\quad -\,0.6901 \\ -\,0.7466 &{}\quad 0.0913 &{}\quad -\,0.6901 &{}\quad -\,5.1909 \end{bmatrix}\\&{\overline{P}}^1_{D_2} = \begin{bmatrix} 0.1731 &{}\quad 0 &{}\quad 1.8992 &{}\quad -\,0.5194 \\ 0 &{}\quad 1.0586 &{}\quad 0 &{}\quad -\,0.6881 \\ 1.8992 &{}\quad 0 &{}\quad -\,0.2317 &{}\quad 3.8167 \\ -\,0.5194 &{}\quad -\,0.6881 &{}\quad 3.8167 &{}\quad 0 \end{bmatrix} \\&{\overline{P}}^1_{D_3} = \begin{bmatrix} 0.2126 &{}\quad 0 &{}\quad -\,0.1039 &{}\quad -\,0.9511 \\ 0 &{}\quad 0.9163 &{}\quad 0 &{}\quad 0.1315 \\ -\,0.1039 &{}\quad 0 &{}\quad -\,0.1288 &{}\quad -\,0.2569 \\ -\,0.9511 &{}\quad 0.1315 &{}\quad -\,0.2569 &{}\quad -\,6.1378 \end{bmatrix}\\&{\overline{P}}^1_{D_4} = \begin{bmatrix} 1.3207 &{}\quad 0 &{}\quad 1.4462 &{}\quad -\,0.8 \\ 0 &{}\quad 0.9395 &{}\quad 0 &{}\quad -\,0.0918 \\ -\,1.4462 &{}\quad 0 &{}\quad -\,1.2602 &{}\quad 0.2270 \\ -\,0.8 &{}\quad -0.0918 &{}\quad 0.2270 &{}\quad 0 \end{bmatrix} \\&{\overline{P}}^1_{D_5} = \begin{bmatrix} 0.2126 &{}\quad 0 &{}\quad 0 &{}\quad -\,1.055 \\ 0 &{}\quad 0.7874 &{}\quad 0 &{}\quad -\,3.1943 \\ 0 &{}\quad 0 &{}\quad 0 &{}\quad 0 \\ -\,1.055 &{}\quad -\,3.1943 &{}\quad 0 &{}\quad 0 \end{bmatrix}\\&{\overline{P}}^1_{D_6} = \begin{bmatrix} 0.2126 &{}\quad 0 &{}\quad 0 &{}\quad 0.5095 \\ 0 &{}\quad 0.7874 &{}\quad 0 &{}\quad -\,3.1943 \\ 0 &{}\quad 0 &{}\quad 0 &{}\quad 0 \\ 0.5095 &{}\quad -\,3.1943 &{}\quad 0 &{}\quad -\,3.1289 \end{bmatrix} \\&{\overline{P}}^1_{D_7} = \begin{bmatrix} 0.2126 &{}\quad 0 &{}\quad 0 &{}\quad -\,1.055 \\ 0 &{}\quad 0.7874 &{}\quad 0 &{}\quad 0.4160 \\ 0 &{}\quad 0 &{}\quad 0 &{}\quad 0 \\ -1.055 &{}\quad 0.4160 &{}\quad 0 &{}\quad -\,7.2205 \end{bmatrix}\\&{\overline{P}}^1_{D_8} = \begin{bmatrix} 0.2126 &{}\quad 0 &{}\quad 0 &{}\quad 0.5095 \\ 0 &{}\quad 0.7874 &{}\quad 0 &{}\quad 0.4160 \\ 0 &{}\quad 0 &{}\quad 0 &{}\quad 0 \\ 0.5095 &{}\quad 0.4160 &{}\quad 0 &{}\quad -\,10.3493 \end{bmatrix} \end{aligned}$$
